# *Cis*- and *trans*-action of the cold-induced lncRNAs, *SVALKA* and *SVALNA*, regulate *CBF1* and *CBF3* in Arabidopsis

**DOI:** 10.1038/s44319-025-00568-5

**Published:** 2025-09-01

**Authors:** Isabell Rosenkranz, Sarah Mermet, Vasiliki Zacharaki, Peter Kindgren

**Affiliations:** Umeå Plant Science Centre, Department of Forest Genetics and Plant Physiology, Swedish University of Agricultural Sciences, 90187 Umeå, Sweden

**Keywords:** Arabidopsis, Cold Response, Epigenetic Regulation, Long Non-coding RNAs, Plant Biology, RNA Biology

## Abstract

Long noncoding RNAs (lncRNAs) are emerging as key regulatory players of coding gene expression in eukaryotes. Here, we investigate the roles of the lncRNAs *SVALKA (SVK)* and *SVALNA (SVN)* in regulating *CBF1* and *CBF3* gene expression in Arabidopsis under cold stress conditions. We integrated omics approaches, together with genetics and molecular biology, to uncover the transcriptional dynamics and regulatory mechanisms of *SVK* and *SVN*. Our results demonstrate that *SVK* functions as a *cis*- and *trans*-acting lncRNA, regulating both *CBF1* and *CBF3* through RNAPII collision and chromatin remodeling, while *SVN* serves a *cis* role by negatively regulating *CBF3* via a RNAPII collision mechanism. We identified isoforms of *SVK*, originating from distinct transcription start sites and undergo alternative splicing which might be important to adapt stability, crucial for the regulatory functions. Furthermore, we show that two positionally conserved lncRNAs, originating from the upstream antisense strand of neighboring genes, can have different molecular mechanisms to regulate their targets. This study elucidates the complex interplay of lncRNAs in gene regulation, highlighting their essential roles in modulating responses to environmental stresses. Our findings contribute to a deeper understanding of the mechanisms underlying lncRNA functionality and their significance in gene regulatory networks in eukaryotes.

## Synopsis

The lncRNAs *SVALKA* and *SVALNA* negatively regulate *CBF* gene expression in *Arabidopsis* during cold stress. Modes of action include RNA Polymerase II collision mechanisms, Transcription start site (TSS) switch and splicing as well as recruitment of the PRC2 protein complex. This study highlights the complex regulation and molecular mechanisms that lncRNAs employ to regulate their target genes.

- *SVK* and *SVN* function in cis regulating *CBF1* and *CBF3* via an RNA Polymerase II collision mechanism and remodeling of chromatin.
- A *SVK* isoform, originating from a TSS switch, undergoes splicing and recruits PRC2 to *CBF3*.
- Regulation of *CBF*s by *SVK* and *SVN* is crucial for cold stress adaptation, showcasing the importance of lncRNAs in the cold response in *Arabidopsis*.

## Abstract (web summary)

The lncRNAs SVALKA and SVALNA negatively regulate CBF gene expression in Arabidopsis during cold stress. Modes of action include RNA Polymerase II collision mechanisms, Transcription start site (TSS) switch and splicing as well as recruitment of the PRC2 protein complex. This study highlights the complex regulation and molecular mechanisms that lncRNAs employ to regulate their target genes.

## Graphical abstract

**Figure:**
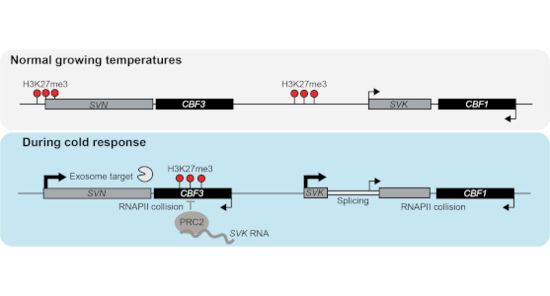

**Figure:**
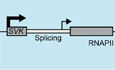

## Introduction

Long noncoding RNAs (lncRNAs) are a diverse class of RNA molecules longer than 200 nucleotides that play essential roles in gene regulation (Rinn and Chang, 2012; Zhao et al, 2018). In contrast to small RNAs, which primarily engage in RNA interference pathways, lncRNAs function through various mechanisms such as acting as molecular scaffolds, decoys, guides, or enhancers involved in transcriptional and post-transcriptional processes (Geisler and Coller, 2013; Kashi et al, 2016). They influence key biological functions by interacting with DNA, RNA, and proteins, thereby modulating chromatin dynamics, transcription factor activity, and RNA stability (Csorba et al, 2014; Wang et al, 2011; Wilusz et al, 2009).

In plants, lncRNAs contribute to key developmental processes such as flowering (Csorba et al, 2014; Kim et al, 2017), hormone signaling (Ariel et al, 2014), and responses to abiotic stresses (Gómez**-**Martínez et al, 2023; Kindgren et al, 2018). By interacting with chromatin-modifying complexes, such as Polycomb Repressive Complex 2 (PRC2), lncRNAs can direct histone modifications to specific loci, thereby fine-tuning gene expression (Brockdorff, 2013). For instance, at the *FLOWERING LOCUS C* (*FLC*) gene locus in *Arabidopsis thaliana*, several lncRNAs have been proposed to recruit PRC2, via binding of the PRC2 subunit CLF, to deposit the repressive histone mark H3K27me3 (Heo and Sung, 2011; Kim et al, 2017). Another lncRNA, *COOLAIR*, functions in parallel, and in a PRC2-independent mechanism, to remove the active H3K36me3 mark from the *FLC* gene body (Nielsen et al, 2024). The activity of two distinct repressive mechanisms is believed to give the plant flexibility and temporal plasticity to regulate *FLC* output in response to cold (Nielsen et al, 2024).

As sessile organisms, plants must rely on intricate regulatory networks to perceive and respond to fluctuating environmental conditions (Urano et al, 2010). A well-characterized pathway involved in cold stress adaptation is the CBF-dependent pathway, in which C-repeat/dehydration-responsive element binding factors (*CBF*s) serve as central regulators for cold acclimation (Gilmour et al, 1998; Jaglo**-**Ottosen et al, 1998). In Arabidopsis, the *CBF1-3* genes are arranged in tandem on chromosome 4 and are tightly controlled during cold stress to enhance plant survival (Gilmour et al, 1998; Medina et al, 1999). Like *FLC*, *CBF1*, and *CBF3* are negatively regulated by lncRNAs. *SVALKA* (*SVK*), an antisense lncRNA transcribed downstream of *CBF1*, fine-tunes the expression of both *CBF1* and *CBF3*, albeit with distinct mechanisms (Gómez**-**Martínez et al, 2023; Kindgren et al, 2018). Upon 2–4 h of low-temperature exposure, RNA Polymerase II (RNAPII) read-through transcription of a short *SVK* α isoform leads to head-to-head RNAPII collisions within the *CBF1* gene body. These collisions trigger premature termination of *CBF1* transcription and degradation of the transcript, ultimately repressing *CBF1* expression (Kindgren et al, 2018). Under normal growing conditions (22 °C), a longer isoform of *SVK* α (*SVK-L*) functions as a natural antisense transcript to *CBF1*, reducing *CBF1* mRNA levels by forming double-stranded RNA that is subsequently degraded (Zacharaki et al, 2023). In addition, a longer isoform (*SVK* β) originating from a distal TSS of *SVK* interacts with CLF to deposit H3K27me3 marks over the *CBF3* gene body after prolonged exposure to low temperatures (Gómez**-**Martínez et al, 2023). The dynamic RNA regulation of *SVK* and its various isoforms are essential for its regulatory functions, allowing the lncRNA to engage with multiple molecular partners and participate in complex gene networks.

Here, we identify and characterize a novel long noncoding RNA *SVALNA*, transcribed downstream of *CBF3*. Unlike *SVK*, *SVN* is a target for the nuclear exosome and is rapidly degraded, confining it to a negatively regulated *cis* role on *CBF3* by RNAPII collision in a temporal and mechanistic manner akin to the *SVK-CBF1* circuit. This suggests a complex dynamic where *SVN* serves as a critical regulatory element that fine-tunes *CBF3* expression prior to epigenetic repression by *SVK*. Furthermore, we show that the *trans* role of *SVK* on *CBF3* depends on an epigenetically controlled switch from a proximal to a distal TSS and splicing. Thus, our research highlights the active involvement and temporal regulation of distinct isoforms of lncRNAs to achieve regulatory flexibility in response to changing environmental conditions.

## Results

### A rapidly degraded long noncoding RNA, *SVALNA*, is transcribed downstream of *CBF3*

**Figure 1:**
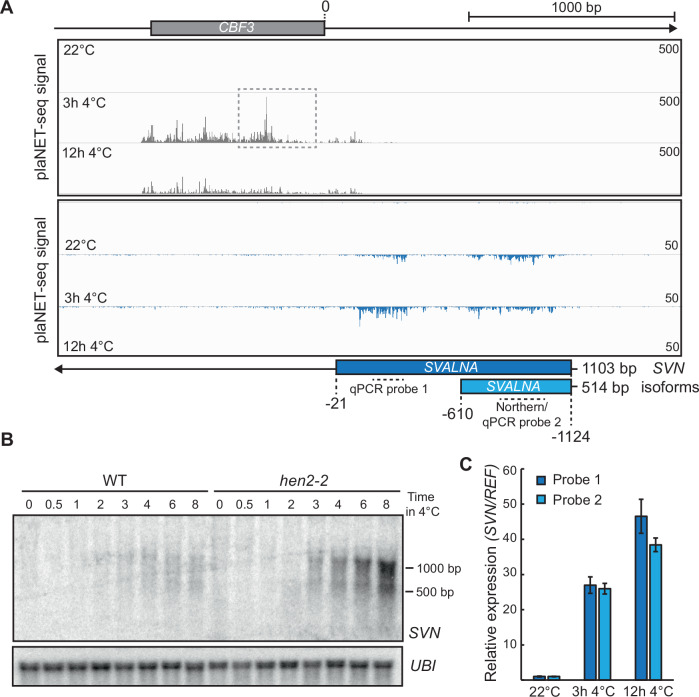
Characterization of the lncRNA *SVALNA.* (**A**) plaNET-seq signal after 3 h and 12 h at 4 °C and in control conditions at the *CBF3* and *SVN* locus. Nascent RNAPII transcription is shown for sense and antisense transcripts in gray and blue, respectively. Below the screenshot, the *SVN* isoforms are shown. The start and end of the isoforms are relative to the Poly(A) signal of *CBF3*. The binding sites of the Northern/qPCR probes are indicated as dashed lines. (**B**) Northern blot of a cold exposure time series of Col-0 (WT) and *hen2-2*. The probe used for *SVN* is shown in (**A**). Blots were repeated with three biological replicates. *UBI* was used as loading control. (**C**) Relative *SVN* isoform expression determined by RT-qPCR in WT. Bars represent mean ± SEM from three biological replicates. The relative level of *SVN* transcripts were normalized to the level in WT in control conditions. Source data are available online for this figure.

At the *CBF3* locus in Arabidopsis, we detected a long noncoding RNA (hereafter named *SVALNA*, *SVN*) with available plant Native Elongation Transcript sequencing (plaNET-seq) (Fig. 1A) (Kindgren et al, 2020). *SVN* was upregulated by cold and reached higher transcriptional activity later in the cold response compared to *CBF3*. We also detected RNAPII stalling after 3 h at 4 °C at the locus (Fig. 1A). *SVN* is barely detected in wild-type but clearly accumulates in a nuclear exosome mutant, *hua enhancer 2-2* (*hen2-2*) (Fig. 1B). This suggests that *SVN* is a target for rapid degradation by the nuclear exosome and further indicates that *SVN* transcription is more important than the *SVN* RNA product. To characterize transcription events along *SVN*, we used available transcription start sequencing data in wild-type and *hen2-2* (Thomas et al, 2020) to detect the main initiation site (1124 bp downstream of the poly(A) site of *CBF3*) (Fig. EV1A). With publicly available direct RNA-sequencing data (Schurch et al, 2014), we detected two clusters of end sites for *SVN* (DR-seq, Fig. EV1A), which corresponded to the two isoforms (514 bp and 1103 bp) detected by our Northern blot analysis (Figs. 1B and EV1A). Further confirmation could be obtained by publicly available RNA-seq data from wild-type and *hen2-2* (Fig. EV1B) (Bhat et al, 2024). In addition, we confirmed the two isoforms with available Transcription isoform sequencing (TIF-seq, Fig. EV1C) (Thomas et al, 2020). The longer isoform was the dominant one later compared to earlier in the cold response (Fig. 1B) and its poly(A) site was only 21 bp from the main poly(A) site of *CBF3* (Fig. EV1A). We confirmed the expression pattern of the isoforms of *SVN* with RT-qPCR (Fig. 1C). Taken together, *SVN* is an unstable long noncoding RNA transcribed downstream of *CBF3* on the antisense strand. Further, *SVN* has a potential regulatory role of *CBF3* based on RNAPII stalling patterns along the *SVN-CBF3* region.

**Figure EV1:**

Detection and characterization of *SVN* transcripts. (**A**) plaNET-seq coverage profile for *SVN* with positions of RNAPII are shown for the sense strand in blue. TSS-seq (transcription start site sequencing) for WT and *hen2-2* and DR-seq (direct RNA sequencing) for WT tracks are also shown. The DR-seq track reveals sites of mRNA cleavage and polyadenylation (PAS). (**B**) Screenshot of the *SVN* locus from an RNA-seq dataset. Shown are WT and *hen2-2* at 22 °C and after 12 h at 4 °C. Elevated transcriptional activity is indicated by higher peak density and amplitude. (**C**) Screenshot of the *SVN* locus from a TIF-seq dataset. Shown are WT and *hen2-2* after 3 h at 4 °C.

### The long noncoding RNA, *SVALKA*, is relatively stable and has a complex transcriptional regulation

**Figure 2:**
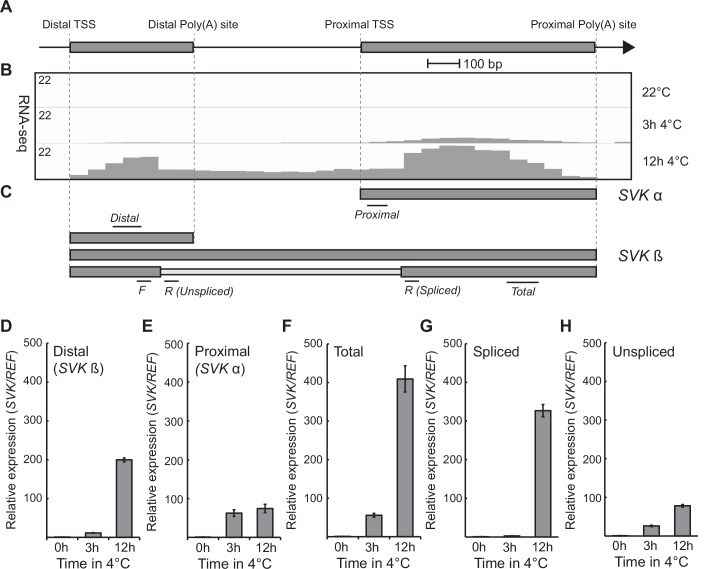
Characterization of *SVK* isoforms. (**A**) Graphical representation of TSS and Poly(A) signal of *SVK*. (**B**) Screenshot of the *SVK* locus from an RNA-seq dataset. Elevated transcriptional activity is indicated by higher peak density and amplitude. (**C**) Graphical representation of *SVK* isoforms and positions of qPCR probes used to identify relative expression of isoforms. (**D**–**H**) Relative expression of *SVK* isoforms determined by RT-qPCR in WT during cold exposure. The distal (**D**), proximal (**E**), total (**F**), spliced (**G**), and unspliced (**H**) transcripts of *SVK* were analyzed. Bars represent mean ± SEM from three biological replicates. The relative level of *SVK* transcripts was normalized to the level in control conditions. Source data are available online for this figure.

The rapid degradation of *SVN* raises the question of how the previously studied lncRNA, *SVALKA (SVK)*, which regulates both the neighboring gene *CBF1* as well as *CBF3*, achieves relatively higher stability. *SVK* has two TSS and two poly(A) signals (Kindgren et al, 2018; Zacharaki et al, 2023). A group of shorter transcripts (*SVK* α) is initiated from a proximal TSS (in relation to *CBF1*) (Fig. 2A) while transcripts from a distal TSS (*SVK* β) are either terminated at a distal poly(A) site or may undergo splicing and continue to the proximal poly(A) site (Fig. 2A–C). Both *SVK* α transcripts and spliced *SVK* β have a length of around 750 bp, making them difficult to separate on a northern blot with a probe annealing to the 3ʹ-end of *SVK* (Kindgren et al, 2018). In available RNA-seq data, it was clear that the proximal TSS was more active early in the cold response (3 h 4 °C) while the distal TSS was activated later (12 h 4 °C) (Fig. 2B), corroborating earlier studies (Kindgren et al, 2018). Thus, to disentangle the active forms of *SVK*, we designed specific RT-qPCR oligos that amplify only *SVK* α or *SVK* β (Fig. 2C). In addition, we designed oligos that would amplify all *SVK* transcripts (Total) and spliced and unspliced version of *SVK* β (Fig. 2C). Our RT-qPCR analysis confirmed that the distal TSS is activated later than the proximal TSS and that it is foremost the spliced isoform of *SVK* β that accumulates later in the cold response (12 h 4 °C) (Fig. 2D–H). Interestingly, the activation of *SVN* and the distal TSS of *SVK* are correlated to changes in the chromatin environment, specifically around the promoter which is shown in a publicly available ChIP-seq dataset of 21-day-old seedlings grown under short-day conditions (Fig. EV2A,B). In this dataset, a peak of the repressive mark, H3K27me3, could be detected at control conditions at both promoters. This peak rapidly decreased in amplitude following cold treatment (3 h and 72 h of 4 °C) while the activation mark, H3K4me3 increases (Fig. EV2A,B). These results indicate that *SVK* switches TSS, from a proximal to a distal start site during the cold response. Both the activation of *SVN* and *SVK* β is regulated by the chromatin environment at the respective promoter.

**Figure EV2:**

Histone marks along the gene body of *SVK* and *SVN.* (**A**) Screenshot of the *SVK* locus from a ChIP-seq dataset. Shown are WT at 22 °C and after 3 h and 72 h at 4 °C. Higher occupancy of H3K4me3 and H3K27me3 are indicated by higher peak density and amplitude. (**B**) Screenshot of the *SVN* locus from a ChIP-seq dataset. Shown are WT at 22 °C and after 3 h and 72 h at 4 °C. Higher occupancy of H3K4me3 and H3K27me3 are indicated by higher peak density and amplitude.

**Figure 3:**
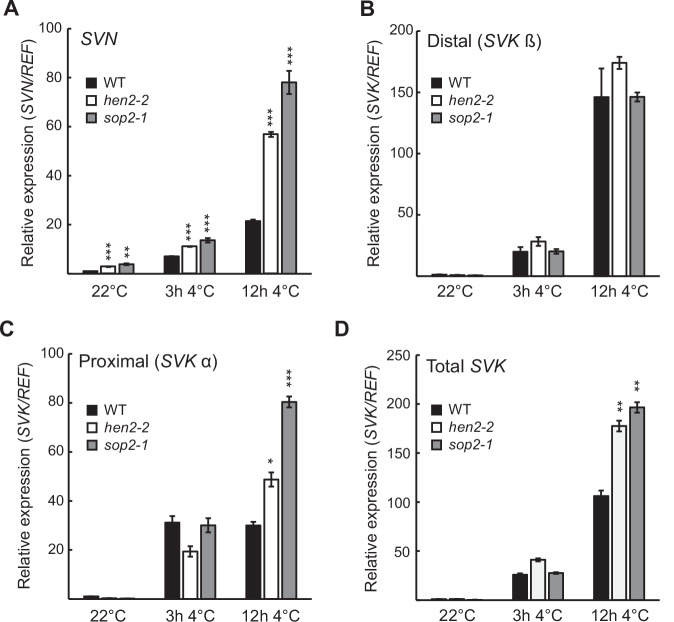
Nuclear exosome sensitivity of *SVN* and *SVK* isoforms. (**A**) Relative expression of *SVN* determined by RT-qPCR in WT and exosome mutants during cold conditions. Bars represent mean ± SEM from three biological replicates. The relative level of *SVN* was normalized to the level in WT in control conditions. Statistically significant differences between means were calculated with Student’s *t* test (*P < 0.05, **P < 0.01, ***P < 0.001). Exact *P* values for *hen2-2* were *P* = 0.000096 (22 °C), *P* = 0.000098 (3 h 4 °C), and *P* = 0.000026 (12 h 4 °C). For *sop2-1*, exact *P* values were *P* = 0.0050 (22 °C), *P* = 0.0093 (3 h 4 °C), and *P* = 0.00095 (12 h 4 °C). All *P* values can be found in the source data. (**B**–**D**) Relative expression of *SVK* isoforms determined by RT-qPCR in WT and exosome mutants that affect the expression of *SVK* during cold conditions. The distal (**B**), proximal (**C**), and total (**D**) transcript of *SVK* were analyzed. Bars represent mean ± SEM from three biological replicates. The relative level of *SVK* transcripts were normalized to the level in WT in control conditions. Statistically significant differences between means were calculated with Student’s *t* test (*P < 0.05, **P < 0.01, ***P < 0.001). Exact p values for *hen2-2* were *P* = 0.013 (**C**), and *P* = 0.0029 (**D**). For *sop2-1*, exact *P* values were *P* = 0.00021 (**C**), and *P* = 0.0010 (**D**). All *P* values can be found in the source data. Source data are available online for this figure.

To confirm the rapid degradation of *SVN*, and to investigate if the different isoforms of *SVK* were rapidly degraded by the nuclear exosome, we used *sop2-1* and *hen2-2* mutants. The *sop2-1* allele has a point mutation in the RRP4 exosome subunit and accumulates all exosome targets (Hematy et al, 2016). *SVN* accumulated in both mutants, corroborating our earlier results (Fig. 3A). For the *SVK* isoforms, only the *SVK* α transcripts were accumulating in the mutants (Fig. 3B,C), leading to an overall accumulation of *SVK* transcripts (Fig. 3D). The accumulation of *SVK* transcripts only occurred after 12 h 4 °C, indicating that most amplicons represent unspliced forms of *SVK* β. We confirmed that this was the case by amplifying spliced and unspliced *SVK* β in the exosome mutants (Fig. EV3A,B). Combined, our results show major differences in the regulation of *SVN* and *SVK*. While both transcripts show a similar expression pattern, *SVN* is unstable relative to *SVK*, and the switch of TSS and the introduction of a splicing event of *SVK*, but not for *SVN*, may be important for *SVK* function and stability.

**Figure EV3:**

Nuclear exosome sensitivity of spliced and unspliced *SVK* β. (**A**, **B**) Relative spliced (**A**) and unspliced (**B**) *SVK* expression determined by RT-qPCR in WT, *hen2-2* and *sop2-1* mutants during cold conditions. Bars represent mean ± SEM from three biological replicates. The relative levels of transcripts were normalized to the level in WT in control conditions. Statistically significant differences between means were calculated with Student’s *t* test (***P < 0.001). Exact *P* value for *hen2-2* was *P* = 0.0023. For *sop2-1*, exact *P* value was *P* = 0.00082.

### A TSS switch and splicing of *SVK* are essential for its *trans*-acting role

**Figure 4:**
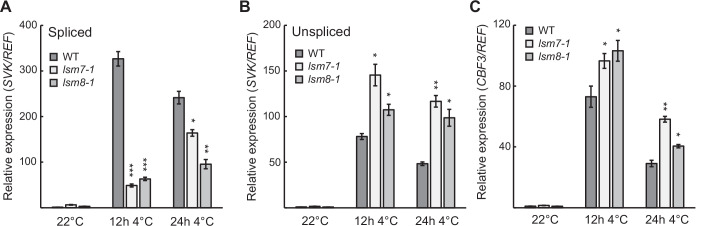
Splicing of *SVK.* (**A**–**C**) Relative expression of the spliced *SVK* (**A**) and unspliced *SVK* (**B**) isoforms and *CBF3* (**C**) determined by RT-qPCR in WT and mutants that affect splicing during cold exposure. Bars represent mean ± SEM from three biological replicates. The relative level of *SVK* transcripts was normalized to the level in WT in control conditions. Statistically significant differences between means were calculated with Student’s *t* test (*P < 0.05, **P < 0.01, ***P < 0.001). Exact *P* values for *lsm7-1* were (**A**) *P* = 0.00023 (12 h 4 °C), and *P* = 0.037 (24 h 4 °C); (**B**) *P* = 0.018 (12 h 4 °C), and *P* = 0.0027 (24 h 4 °C); (**C**) *P* = 0.042 (12 h 4 °C), and *P* = 0.00232 (24 h 4 °C). For *lsm8-1*, exact *P* values were (**A**) *P* = 0.00029 (12 h 4 °C), and *P* = 0.0047 (24 h 4 °C); (**B**) *P* = 0.046 (12 h 4 °C), and *P* = 0.022 (24 h 4 °C); (**C**) *P* = 0.049 (12 h 4 °C), and *P* = 0.027 (24 h 4 °C). All *P* values can be found in the source data. Source data are available online for this figure.

**Figure 5:**
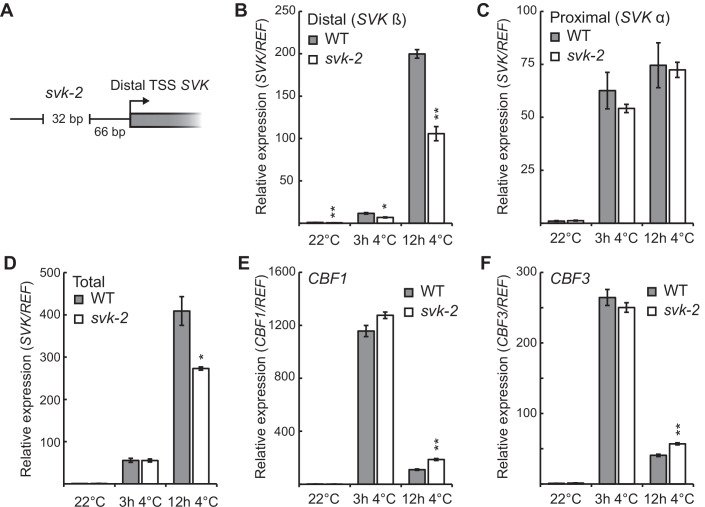
Characterization of *SVK* CRISPR-Cas9 deletion line *svk-2.* (**A**) Graphical representation of the position of the deletion in the line *svk-2* induced by CRISPR-Cas9 used in this study. (**B**–**D**) Relative expression of *SVK* isoforms determined by RT-qPCR in WT and *svk-2* that affect the expression of *SVK* during cold conditions. The distal (**B**), proximal (**C**) and total (**D**) transcript of *SVK* were analyzed. Bars represent mean ± SEM from three biological replicates. The relative level of *SVK* transcripts was normalized to the level in WT in control conditions. Statistically significant differences between means were calculated with Student’s *t* test (*P < 0.05, **P < 0.01). For *svk-2*, exact *P* values were (**B**) *P* = 0.042 (3 h 4 °C), and *P* = 0.0020 (12 h 4 °C); (**D**) *P* = 0.039 (12 h 4 °C). All *P* values can be found in the source data. (**E**, **F**) Relative *CBF1* (**E**) and *CBF3* (**F**) expression determined by RT-qPCR in WT and *svk-2* during cold conditions. Bars represent mean ± SEM from three biological replicates. The relative level of *CBF1* and *CBF3* transcripts were normalized to the level in WT in control conditions. Statistically significant differences between means were calculated with Student’s *t* test (*P < 0.05, **P < 0.01). For *svk-2*, exact *P* values were (**E**) *P* = 0.0063 (12 h 4 °C); (**F**) *P* = 0.0071 (12 h 4 °C). All *P* values can be found in the source data. Source data are available online for this figure.

**Figure EV4:**
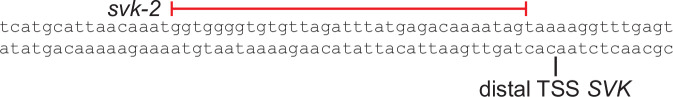
Characterization of *SVK* CRISPR-Cas9 deletion line *svk-2.* Graphical representation of the position of the deletion in the lines *svk-2* induced by CRIPSR-Cas9 used in this study.

To test the involvement of splicing in the maturation of *SVK*, we used two mutants of the spliceosome, *sm-like 7-1* and *8-1* (*lsm7-1* and *lsm8-1*). Both mutants are part of the nucleoplasmic version of the spliceosome (Nardeli et al, 2023; Perea**-**Resa et al, 2012). We used longer exposure to cold in these experiments (12 and 24 h 4 °C) to investigate the reported *trans*-role for *SVK* on *CBF3* (Gómez**-**Martínez et al, 2023). The spliced version of *SVK* was greatly reduced in the two mutants, while the unspliced version was increased (Fig. 4A,B). This splicing mis-regulation of *SVK* had a significant effect on the repressive role of *CBF3* (Fig. 4C), indicating that splicing is essential for the *trans* role of *SVK*. These results suggest that the splicing machinery is crucial for the mode-of-action of *SVK* on *CBF3*. To further show the significance, we obtained CRISPR-Cas9-mediated deletion lines targeting the promoter of the distal TSS of *SVK*. A stable line designated as *svk-2*, with a 32 bp deletion, 66 bp upstream of the distal TSS was generated (Figs. 5A and EV4). In *svk-2*, only the distal transcription was affected (Fig. 5B,C). This leads to an overall lower *SVK* level after 12 h 4 °C (Fig. 5D). Furthermore, the peak of *CBF1* and *CBF3* expression (3 h at 4 °C) was not mis-regulated in *svk-2*, but the repression of both genes (12 h at 4 °C) was (Fig. 5E,F). Thus, the transcription from the distal TSS of *SVK* is crucial for its repressive role on *CBF1* and *CBF3*. In summary, in contrast to *SVN*, *SVK* function requires several steps of transcriptional regulation, such as TSS switch and splicing.

### Both *SVN* and *SVK* utilize an RNAPII collision mechanism to repress *CBF3* and *CBF1*

**Figure 6:**
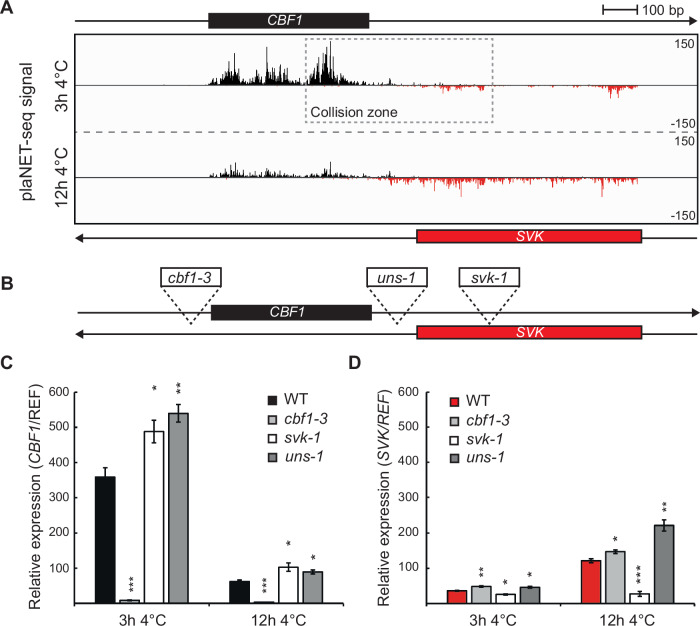
RNAPII collisions can be inferred by restricting expression strand-specifically*.* (**A**) plaNET-seq signal after 3 h and 12 h at 4 °C at the *CBF1* and *SVK* locus. Nascent RNAPII transcription is shown for sense and antisense transcripts in black and red, respectively. The dotted line rectangle delimits the RNAPII collision zone. (**B**) Graphical representation of the insertion positions of the T-DNA lines used in this study. (**C**, **D**) Relative *CBF1* (**C**) and *SVK* total (**D**) expression determined by RT-qPCR in WT, *cbf1* and *svk* mutants during cold exposure. Bars represent mean ± SEM from three biological replicates. The relative level of *CBF1* and *SVK* transcripts was normalized to the level in WT in control conditions. Statistically significant differences between means were calculated with Student’s *t* test (*P < 0.05, **P < 0.01, ***P < 0.001). Exact *P* values for *cbf1-3* were (**C**) *P* = 0.00020 (3 h 4 °C), and *P* = 0.0049 (12 h 4 °C); (**D**) *P* = 0.0096 (3 h 4 °C), and *P* = 0.030 (12 h 4 °C). For *svk-1*, exact *P* values were (**C**) *P* = 0.037 (3 h 4 °C), and *P* = 0.031 (12 h 4 °C); (**D**) *P* = 0.012 (3 h 4 °C), and *P* = 0.00052 (12 h 4 °C). For *uns-1*, exact *P* values were (**C**) *P* = 0.0077 (3 h 4 °C), and *P* = 0.019 (12 h 4 °C); (**D**) *P* = 0.033 (3 h 4 °C), and *P* = 0.0039 (12 h 4 °C). All *P* values can be found in the source data. Source data are available online for this figure.

To elucidate the mode-of-action of *SVN*, we started to visualize the collision zone of *CBF1* and *SVK* that occurs post-peak of *CBF1* expression (after three to eight hours at 4 °C), with plaNET-seq data (Kindgren et al, 2020). Accumulation of actively transcribing RNAPII complexes can be seen on both strands, designating a hallmark for RNAPII collision (Fig. 6A), similar to what we detected at the *CBF3-SVN* locus (Fig. 1A). To further support the collision mechanism, we used a line that harbors a transfer DNA (T-DNA) insertion in the *CBF1* promoter which greatly limits *CBF1* expression (*cbf1-3*, Fig. 6B) (Zacharaki et al, 2023). In T-DNA lines, large inserts of DNA disrupt the genomic context of the insertion site (Alonso et al, 2003). We also included the *uns-1* mutant that uncouples the expression of *CBF1* and *SVK*, and the *svk-1* mutant that knocks out *SVK* (Kindgren et al, 2018). Both mutations lead to increased *CBF1* expression after cold exposure (Kindgren et al, 2018). Our hypothesis was that restriction of RNAPII transcription on one strand should increase the steady state RNA levels of the transcript derived from the other strand due to fewer collision events occurring after cold exposure (Fig. 6B). As expected, the *uns-1* mutant showed an increased steady state RNA levels for both *CBF1* and *SVK* in response to cold whereas the *cbf1-3* mutant displayed a substantially decreased induction of *CBF1* and increased *SVK* levels (Fig. 6C,D). The *svk-1* mutant showed a decreased *SVK* level and a stronger induction of *CBF1* compared to wild-type. This data supports our initial hypothesis that limited transcription on one strand can decrease the frequency of the collision events and increase the number of transcription events on the other strand to reach their preferred polyadenylation site. Thus, the restriction of transcription in a strand-specific manner in the T-DNA lines not only reinforces the collision mechanism at the *CBF1-SVK* regulatory circuit, but also corroborate with the collision events detected by plaNET-seq.

**Figure 7:**
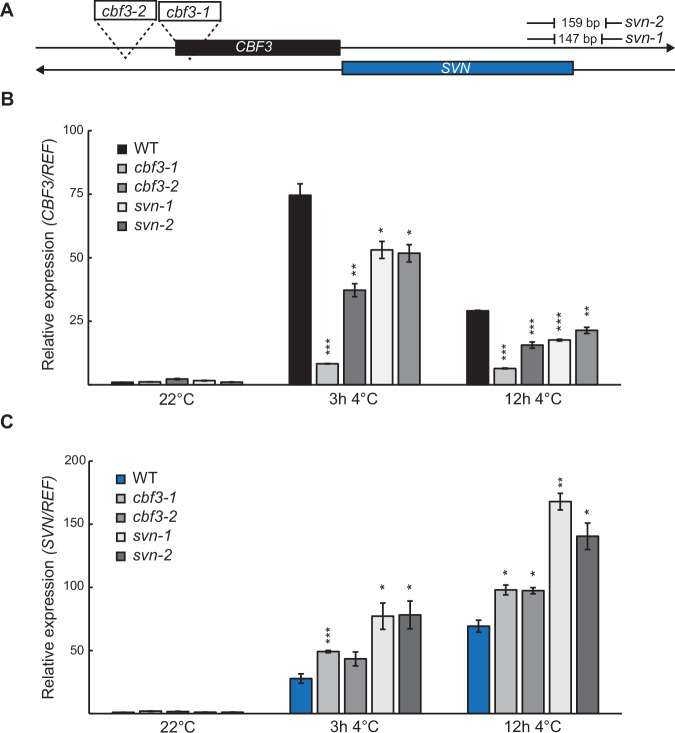
RNAPII collisions occur at the *CBF3-SVN* locus. (**A**) Graphical representation of the insertion positions of the T-DNA lines and deletions induced by CRISPR-Cas9 used in this study. (**B**, **C**) Relative *CBF3* (**B**) and *SVN* (**C**) expression determined by RT-qPCR in WT, *cbf3* and *svn* mutants during cold conditions. Bars represent mean ± SEM from three biological replicates. The relative level of *CBF3* and *SVN* transcripts were normalized to the level in WT in control conditions. Statistically significant differences between means were calculated with Student’s *t* test (*P < 0.05, **P < 0.01, ***P < 0.001). Exact *P* values for *cbf3-1* were (**B**) *P* = 0.00026 (3 h 4 °C), and *P* = 4.2E-07 (12 h 4 °C); (**C**) *P* = 3.9E-06 (3 h 4 °C), and *P* = 0.030 (12 h 4 °C). For *cbf3-2*, exact *P* values were (**B**) *P* = 0.0053 (3 h 4 °C), and *P* = 0.00075 (12 h 4 °C); (**C**) *P* = 0.023 (12 h 4 °C). For *svn-1*, exact *P* values were (**B**) *P* = 0.047 (3 h 4 °C), and *P* = 3.8E-05 (12 h 4 °C); (**C**) *P* = 0.033 (3 h 4 °C), and *P* = 0.0013 (12 h 4 °C). Exact *P* values for *svn-2* were (**B**) *P* = 0.037 (3 h 4 °C), and *P* = 0.0066 (12 h 4 °C); (**C**) *P* = 0.039 (3 h 4 °C), and *P* = 0.012 (12 h 4 °C). All *P* values can be found in the source data. Source data are available online for this figure.

**Figure 8:**
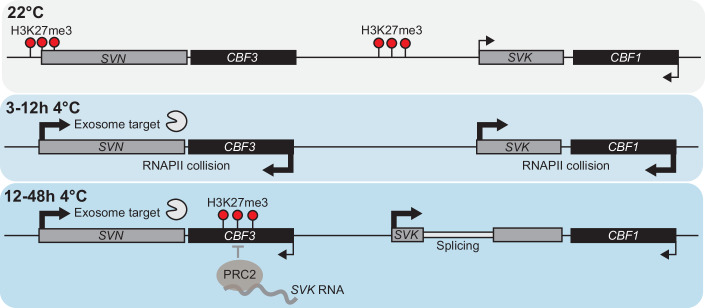
Model of how lncRNAs *SVK* and *SVN* regulate the expression of *CBF1* and *CBF3.* At 22 °C, *CBF* and *SVK* transcription levels are low, with the long *SVK* α isoform forming double-stranded RNA with *CBF1* mRNA, following degradation. *SVN* is quickly degraded by the nuclear exosome. Repressive H3K27me3 marks are present at both loci. Exposure to cold for 3 to 12 h boosts transcription of *CBF* genes and *SVK* and *SVN*, causing premature termination of *CBF1* and *CBF3* mRNA due to RNA polymerase II collisions. Immature mRNA is degraded. During later cold response, *SVK* β becomes the dominant isoform. Splicing of *SVK* β produces a stable transcript that recruits the PRC2 complex, adding repressive marks to *CBF3* and reducing its expression.

**Figure EV5:**
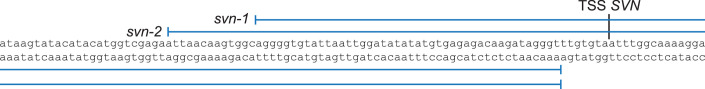
Characterization of *SVN* CRISPR-Cas9 deletion lines *svn-1* and *svn-2.* Graphical representation of the position of the deletion in the lines *svn-1* and *svn-2* induced by CRIPSR-Cas9 used in this study.

To investigate if *SVN* could regulate *CBF3* in a manner similar to the RNAPII collision mechanism, we isolated a T-DNA line in the *CBF3* promoter (*cbf3-2*) and one line in the 5ʹ-UTR (*cbf3-1*) (Fig. 7A). Unfortunately, no T-DNA lines were available at the *SVN* locus. Therefore, we employed a CRISPR-Cas9 deletion strategy to remove the TSS and part of the promoter of *SVN*. Two independent lines were generated, both deleting the *SVN* TSS and part of the promoter (Figs. 7A and EV5). As expected, the *cbf3* mutants both showed a decreased *CBF3* level after exposure to cold (Fig. 7B). This correlated to an increase of *SVN* levels (Fig. 7C), indicating that, indeed, RNAPII collisions are responsible for the regulation of *CBF3* and *SVN* levels in cold conditions. Deletion of the *SVN* promoter in the *svn* mutants resulted in increased *SVN* and decreased *CBF3* steady state levels (Fig. 7B), a corroborating result of the RNAPII collision mechanism. Thus, *SVN* negatively regulates *CBF3* in a similar manner to *SVK* and *CBF1*. In summary, we present an updated model (Fig. 8) showing how the long noncoding RNAs *SVK* and *SVN* regulate *CBF1* and *CBF3*. Under normal conditions (22 °C), *CBF* and *SVK* transcription is low, with the long *SVK* α isoform forming double-stranded RNA with *CBF1* mRNA, leading to cleavage by AGO1 (Zacharaki et al, 2023). During initial cold exposure (within 3 h), transcription of *CBF* genes increases. Later (3–12 h) the expression of the lncRNAs *SVK* and *SVN* increases, causing premature mRNA termination of *CBF1* and *CBF3* due to RNAPII collisions (*cis*-mechanism). A switch in TSS results in *SVK* β becoming dominant later in the cold response. Maturation in the form of *SVK* β splicing produces a stable transcript that recruits the PRC2 complex, adding repressive marks on *CBF3* to suppress its expression (12–24 h, *trans*-mechanism). To conclude, this model highlights the importance of lncRNA isoforms, their stability, and expression patterns in regulating *CBF1* and *CBF3*.

## Discussion

A central theme in our findings is the role of histone modifications, particularly the dynamic levels of repressive H3K27me3 in the *CBF* region. H3K27me3 marks are evicted during cold exposure around the TSS of *SVN* and the distal TSS of *SVK*. This decrease in repressive marks is a well-documented epigenetic mechanism that allows plants to activate genes in response to environmental stimuli (Shen et al, 2021; Yuan et al, 2013). Interestingly, only the distal TSS of *SVK* is decorated with high levels of H3K27me3 at control conditions, not the proximal TSS, explaining the TSS switch to produce *SVK* β when H3K27me3 is removed during the later stages of the cold response. This, in turn, results in PRC2-mediated H3K27me3 enrichment over the *CBF3* gene body by *SVK* β (Gómez**-**Martínez et al, 2023). While the players involved in removing H3K27me3 from the TSS of *SVK* and *SVN* are unknown, there is an established link between lncRNA and PRC2 to add repressive marks to histones. PRC2 regulates numerous targets throughout the genome yet lacking intrinsic DNA-binding affinity. To achieve specificity, PRC2 can form subcomplexes with polycomb-like proteins (PCLs) which help recruit it to unmethylated CpG islands recognized by PCLs (Li et al, 2017). In addition, DNA motifs such as GA-repeats, telobox-like motifs and RY motifs have been shown to assist PRC2 to specific genes (Qüesta et al, 2016; Shu et al, 2020; Wu et al, 2020; Xiao et al, 2017; Yuan et al, 2016; Zhou et al, 2018). Moreover, the interaction between lncRNAs and PRC2 is emerging as a crucial element for balancing gene silencing and activation in eukaryotes (Brockdorff, 2013; Zhao et al, 2018). The role of the PRC2-lncRNA complexes extend beyond a singular case in Arabidopsis; the lncRNAs *COLDAIR* and *COLDWRAP* bound to PRC2 are essential for the repression of the *FLOWERING LOCUS C* (*FLC*) gene during the vernalization process (Heo and Sung, 2011; Kim and Sung, 2017; Kim et al, 2017). Importantly, the PRC2-lncRNA regulatory complex is conserved in other eukaryotes. For example, the human lncRNA *HBL1* interacts with PRC2 in early cardiogenesis by guiding PRC2 occupancy on crucial cardiogenic genes in pluripotent stem cells (Liu et al, 2021). Similarly, the lncRNA *Gm15055* in mouse embryonic stem cells represses the expression of the Hoxa gene by recruiting PRC2 to maintain H3K27me3 at the locus (Liu et al, 2016). In the case of the lncRNA *LEVER* in human cells, the non-polyadenylated, nascent RNA produced by the locus binds and inhibits PRC2 function of the adjacent *β-globin* gene (Teo et al, 2022). The promiscuous binding of RNA to PRC2 might be an important regulatory point for the addition/removal of H3K27me3 at specific gene loci, depending on what RNAs are produced in the vicinity and an avenue for future research. Findings from various eukaryotic organisms underscore a general role for lncRNAs in modulating PRC2 activity, highlighting their importance in the broader context of epigenetic regulation and gene expression control in eukaryotes.

The regulation of the PRC2-*SVK* complex on *CBF3* requires splicing of *SVK*. Thus, the bound transcript (*SVK* β) must possess specific features enabling that interaction. Modular stem-and-loop structures within the RNA have been shown to form unique motifs that allow lncRNAs to bind to proteins (Guttman and Rinn, 2012; Kim et al, 2017; Mercer and Mattick, 2013; Somarowthu et al, 2015, Yang et al, 2022). For instance, *HOTAIR*, a well-studied PRC2-interacting lncRNA in humans, folds into a complex secondary structure that facilitates binding to proteins (Somarowthu et al, 2015). Amongst others, the lncRNA *COOLAIR*, which forms a complex structure with multi-helix junctions, was shown to have an evolutionary conserved secondary structure, making lncRNA transcripts important regulators of gene expression (Corona-Gomez et al, 2023; Hawkes et al, 2016). RNA processing, such as splicing is critical for the functional role of lncRNAs. The lncRNA *HOTAIR* produces three different splice variants. The 5′ end of its longest isoform acts as a scaffold to bind chromatin-modifying complexes (Khan et al, 2023; Tsai et al, 2010; Wu et al, 2013). However, another isoform, generated by alternative splicing, lacks the binding scaffold (Loewen et al, 2014), enabling *HOTAIR* to differentially modulate target genes (Khan et al, 2023). This process emphasizes the pivotal role of alternative splicing of lncRNAs in plants. Kiegle et al, (2018) characterized the lncRNA *LOC9270896* and its isoforms, demonstrating that intron retention and exon skipping yield multiple transcripts crucial for rice seed maturation (Kiegle et al, 2018). The splicing process generates diverse isoforms that can affect RNA stability, localization, and interaction with other molecular partners, thus modulating gene expression (Deveson et al, 2018; Kalsotra and Cooper, 2011; Kiegle et al, 2018). Our findings indicate that the splicing of *SVK* β is crucial for down-regulating *CBF3* following extended cold exposure, as evidenced by measuring gene expression levels in spliceosome mutants (Fig. 4). A decrease in spliced transcript levels leads to elevated *CBF3* expression during cold stress, affirming the significance of the spliced transcript in gene regulation. This raises questions regarding the evolutionary pressures that shape the functional diversity of lncRNAs. Natural antisense lncRNAs can interact via base-pairing (Meena et al, 2024). However, most *SVK* isoforms do not overlap a gene and can serve as scaffolds or guides for chromatin modifiers (Gómez**-**Martínez et al, 2023). They can regulate gene expression in *cis* through transcriptional interference, leading to collisions between RNAPIIs (Kindgren et al, 2018). The stability of *trans*-acting lncRNA isoforms, particularly *SVK*’s stable forms resulting from the use of alternative transcription start sites and splicing, is crucial for its regulatory function. This aligns with findings that stable lncRNAs effectively regulate gene expression, as seen with the mammalian lncRNA *FIRRE*, which has both *cis* and *trans* effects (Fang et al, 2020) and in Arabidopsis where *APOLO* regulates the gene *PID* in *cis* and the *PID* homolog *WAG2* in *trans* by forming R-loops (Ariel et al, 2014; Ariel et al, 2020). The post-transcriptional regulation of lncRNAs adds another layer of complexity for how their function can be controlled to interact with the correct partners at the right time during stress response or development. The lncRNA *APOLO* also interacts with the transcription factor WRKY42 to regulate *RHD6* to induce the growth of root hair when exposed to low temperatures (Moison et al, 2021). Another well-described lncRNA responding to prolonged cold is *VAS*, a lncRNA in winter wheat that stimulates *TaVRN1*, a cold-induced key regulator that is important for floral transition (Xu et al, 2021). Both lncRNAs get activated during low temperatures and recruit transcription factors which lead to the disruption of repressive loops on their target genes, ultimately prompting transcription. *APOLO* and *VAS* are examples of thermosensitive elements whose expression levels change in response to environmental temperature fluctuations. It would be tempting to classify them as thermosensors. A temperature-induced change in their secondary structure, such as a shift in stability or conformation, should, by itself, be sufficient to trigger downstream events. However, this mode-of-action was so far only shown in bacteria (Johansson et al, 2002). Since no clear, direct structure-based sensing by lncRNAs has been observed in eukaryotes, we propose classifying the lncRNAs discussed here, including *SVK* and *SVN*, as thermoresponsive lncRNAs, whose transcriptional activity is regulated by temperature-induced changes in chromatin or other upstream regulatory elements.

In conclusion, our study elucidates the pivotal role of lncRNAs *SVK* and *SVN* in the regulation of *CBF1* and *CBF3* gene expression in response to cold stress. We present an updated model demonstrating that through several mechanisms such as changes in transcription dynamics by RNAPII stalling caused by a switch in isoforms, alternative splicing and recruiting PRC2 complex as well as histone modifications, lncRNAs are crucial in regulating gene expression. The structural characteristics of *SVK* β are essential for its interactions and regulatory functions, emphasizing how splicing generates diverse isoforms that fine-tune gene expression during environmental challenges. Our findings not only advance our understanding of lncRNA functionality but also highlight their fundamental importance as key players in networks that govern gene expression across diverse biological contexts. Ultimately, lncRNAs are not merely regulatory elements, they are one of the cornerstones of complex gene regulation, shaping the biological responses of organisms to their environments.

## Methods

**Table:** Reagents and tools table

Reagent/resource	Reference or source	Identifier or catalog number
**Experimental models**		
*Arabidopsis thaliana/*Col-0		
*Arabidopsis thaliana/* *svk-1*	Kindgren et al, 2018	GABI_145A05
*Arabidopsis thaliana/* *uns-1*		SALK_018442
*Arabidopsis thaliana/cbf3-1*	Khanna et al, 2006	SAIL_244_D02
*Arabidopsis thaliana/* *lsm7-1*	Nardeli et al, 2023	SALK_066076
*Arabidopsis thaliana/* *lsm8-1*	Perea**-**Resa et al, 2012	SALK_048010
*Arabidopsis thaliana/* *cbf3-2*	Nottingham Arabidopsis Stock Centre	GABI_136G01
*Arabidopsis thaliana/* *svk-2*	This study	
*Arabidopsis thaliana/* *svn-1*	This study	
*Arabidopsis thaliana/* *svn-2*	This study	
*E. coli* DH5α		
*A. tumefaciens* GV3101		
**Recombinant DNA**		
pDT1T2	Xing et al, 2014	
pCF588	Christian Fankhauser Lab	
**Antibodies**		
–		
**Oligonucleotides and other sequence-based reagents**		
RT-qPCR and Northern blot primers, genotyping primers and primers used for cloning	This study	Table EV1
**Chemicals, enzymes, and other reagents**		
½ Murashige and Skoog basal medium	Duchefa	Cat #M0221
Sucrose	Duchefa	Cat #S0809
dsDNase	Thermo Fisher Scientific	Cat #EN0771
iScript™ cDNA Synthesis Kit	BioRad	Cat #1708890
iTaq™ Universal SYBR® Green Supermix	BioRad	Cat #1725124
Phusion DNA Polymerase	Thermo Fisher Scientific	Cat #F530S
**Software**		
CHOPCHOP webserver	http://chopchop.cbu.uib.no/	
Microsoft Office programs	Microsoft	
**Other**		
Plant RNA Kit	Omega Biotek	Cat #R6827
CFX384 Real-Time PCR cycler	BioRad	
RNeasy Plant Mini Kit	Qiagen	Cat #74904

### Plant growth and conditions

For the wild-type background, Arabidopsis (*Arabidopsis thaliana*) Col-0 or Columbia accession was employed. For the growth of plants, seeds were surface sterilized and stratified for 2–4 days at 4 °C in the dark and either transferred to soil directly or plated on ½ Murashige and Skoog (MS) basal medium supplemented with 1% (w/v) sucrose. Plants were grown in long day conditions (16 h light, 8 dark, ~100 µE), SciWhite LEDs (Percival) for 10 days. Biological replicates in all experiments represent approximately 20-30 seedlings grown on separate plates. Cold treatment (4 °C, ~25 µE) was initiated at ZT4. T-DNA insertional mutant lines used have been described elsewhere: *svk-1* (GABI_145A05) and *uns-1* (SALK_018442) (Kindgren et al, 2018), *cbf3-1* (SAIL_244_D02) (Khanna et al, 2006), *lsm7-1* (SALK_066076) (Nardeli et al, 2023), *lsm8-1* (SALK_048010) (Perea**-**Resa et al, 2012). The *cbf3-2* (GABI_136G01) mutant was isolated in this study and ordered from the Nottingham Arabidopsis Stock Centre. All T-DNA lines were genotyped and confirmed for homozygosity by PCR. Oligos for genotyping can be found in Table EV1.

### RNA extraction, cDNA synthesis, and RT-qPCR

Total RNA extraction from plant material was carried out using Plant RNA Kit (Omega-Biotek) as per suppliers’ instructions. In total, 1 µg of the extracted RNA was additionally treated with dsDNase (Thermo Fisher Scientific) for the elimination of genomic DNA contamination. Successively, complementary DNA (cDNA) synthesis was carried out using iScript (BioRad) reverse transcriptase as per manufacturer instructions together with a reference gene. Quantitative real-time PCR (RT-qPCR) was performed on CFX384 Real-Time PCR detection systems (BioRad) using SYBR premix (BioRad), cDNA, reverse primer and forward primers at the concentration of 10 pmol/µl with the PCR cycler following initial denaturation at 95 °C for 30 s, standard 40 cycles of 94 °C for 10 s, 60 °C for 30 s. The specificity of RT-qPCR products was assessed from the single peak melt curves. For the data analysis, the Cq values from a minimum of three biological replicates with three technical replicates were averaged and △Cq was obtained as Cq (gene of interest)-Cq (reference gene). Final calculations were performed by following with 2^(-∆cq)^ or 2^(-∆∆cq)^, adjusted to experimentally determined primer efficiency for the determination of fold change in gene expression levels. Statistically significant differences were calculated with two-sided Student’s *t* test. All experiments have been replicated at least two times with independent treatments and samples. Primers used are listed in Table EV1.

### Northern blotting

Total RNA was extracted using RNeasy Plant Mini Kit (Qiagen, Germany) according to the manufacturer’s instructions. In total, 20 µg of total RNA was separated on a 1.2% agarose gel with formaldehyde and 1xMOPS. Gels were blotted overnight onto a nylon membrane and crosslinked with UV radiation. Probes were made in a PCR reaction by incorporating radioactive dTTP (32 P, PerkinElmer, USA) from a DNA probe template. Membranes were exposed to a phosphorimager screen (GE Healthcare, UK) for 1–10 days depending on the expression level of the transcript of interest. Screen was subsequently scanned with a Typhoon scanner (GE Healthcare, UK). The experiments have been replicated with three biological replicates and three blots.

### Generation of CRISPR-Cas9 deletion lines

Guide RNAs (gRNAs) for the generation of CRISPR-Cas9 deletion lines *svk-2*, *svn-1* and *svn-2* were designed using the CHOPCHOP webserver (http://chopchop.cbu.uib.no/). A 2gRNA fragment was amplified utilizing the DT1T2 plasmid (Xing et al, 2014) using Phusion DNA polymerase (Thermo Fisher Scientific). The oligonucleotides used are listed in Table EV1. The resulting PCR product was separated on a gel, excised, purified, and cloned into the targeting vector CF588 (kindly provided by Markus Schmid lab). The modified CF588 vector stems from the pHSE401 binary vector (Xing et al, 2014). In the CF588 vector the antibiotic resistance cassette was replaced with two gRNAs and a seed coat GFP expression to enable faster screening. Final plasmids were validated by sequencing and introduced into WT Col-0 plants via *Agrobacterium tumefaciens* (GV3101) floral dip. T1 seeds were initially screened for GFP expression and subsequently genotyped by PCR. In the T2 generation, seeds lacking GFP fluorescence were selected, and homozygous plants were confirmed by PCR and sequencing for genomic deletion. Seeds from confirmed homozygous plants were used for subsequent experiments.

### Genome-wide datasets

Available genome-wide datasets used in this study include plaNETseq (GSE131733) (Kindgren et al, 2020), RNA-seq (PRJEB67912) (Bhat et al, 2024), TSS-seq and TIF-seq (GSE129523) (Thomas et al, 2020) were analyzed according to the respective papers. The DRS-seq (PRJEB3993) (Schurch et al, 2014) was analyzed according to Kindgren et al, (2020), H3K27me3 and H3K4me3 ChIP-seq bigwig files were used for screenshots (GSE255445) (Faivre et al, 2024).

## Supplementary information

Table EV1

Peer Review File

Source data Fig. 1

Source data Fig. 2

Source data Fig. 3

Source data Fig. 4

Source data Fig. 5

Source data Fig. 6

Source data Fig. 7

Expanded View Figures

## Data Availability

This study includes no data deposited in external repositories. The source data of this paper are collected in the following database record: biostudies:S-SCDT-10_1038-S44319-025-00568-5.
