# Supplementary figures and images for: Cis- and trans-action of the cold-induced lncRNAs, SVALKA and SVALNA, regulate CBF1 and CBF3 in Arabidopsis
Source: EMBO Rep. 2025 Sep 1;26(20):5070–87. doi: 10.1038/s44319-025-00568-5 (PMC12549994; doi:10.1038/s44319-025-00568-5)

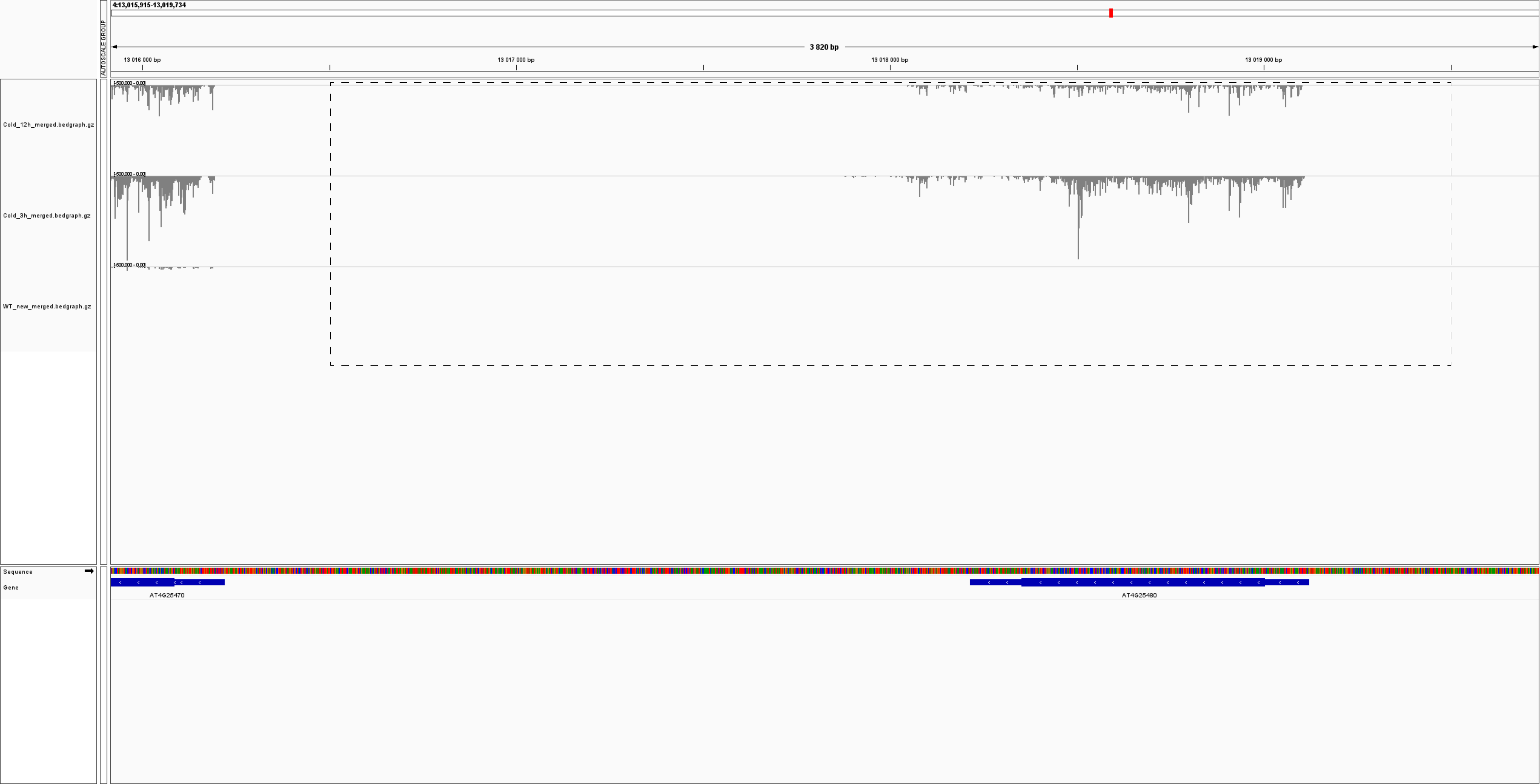

Supplement: Supplementary file 3 — Source data Fig. 1 [file 44319_2025_568_MOESM3_ESM.zip › Figure 1/1A screenshot/plaNETseq CBF3 sense.pdf]

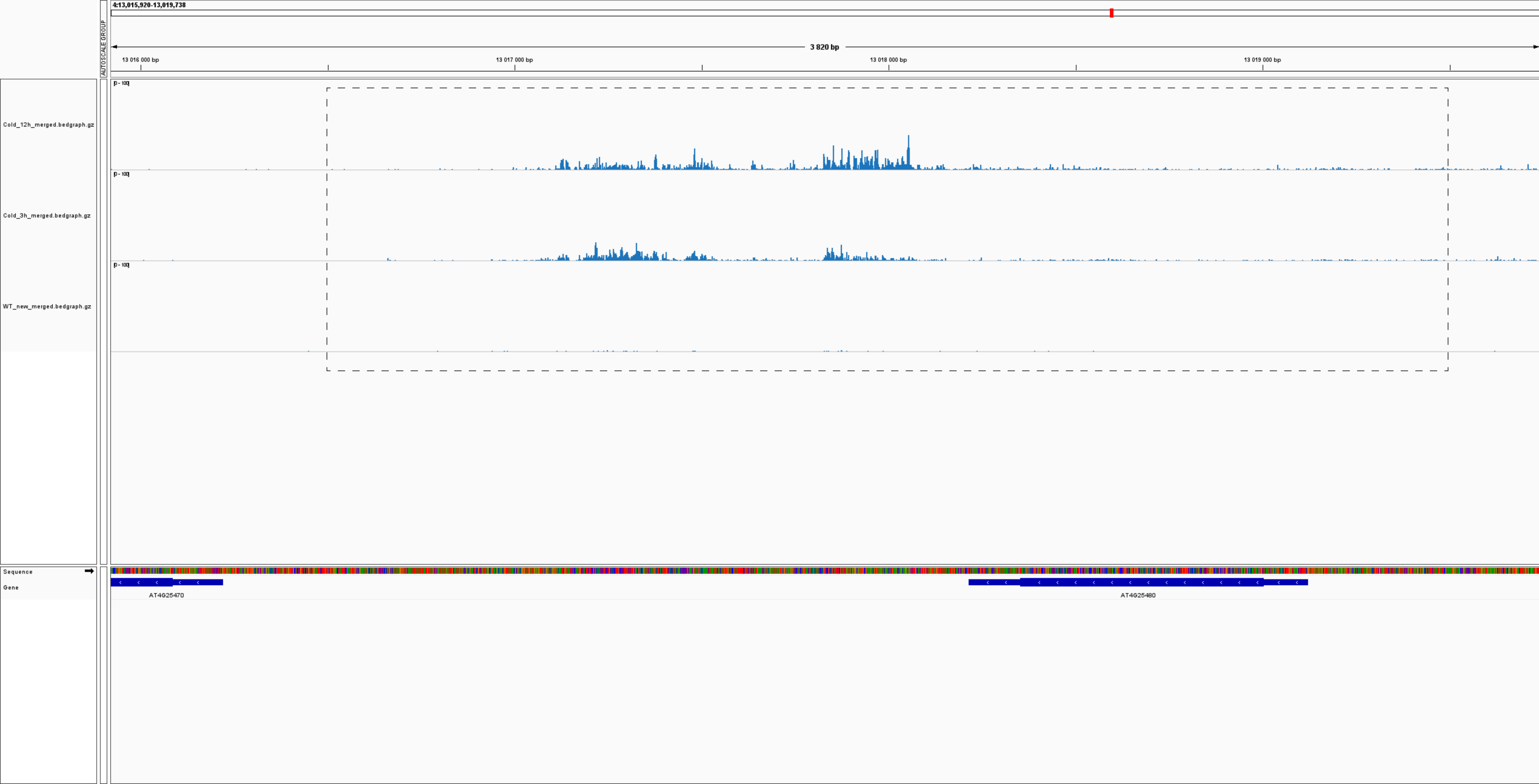

Supplement: Supplementary file 3 — Source data Fig. 1 [file 44319_2025_568_MOESM3_ESM.zip › Figure 1/1A screenshot/plaNETseq SVN antisense.pdf]

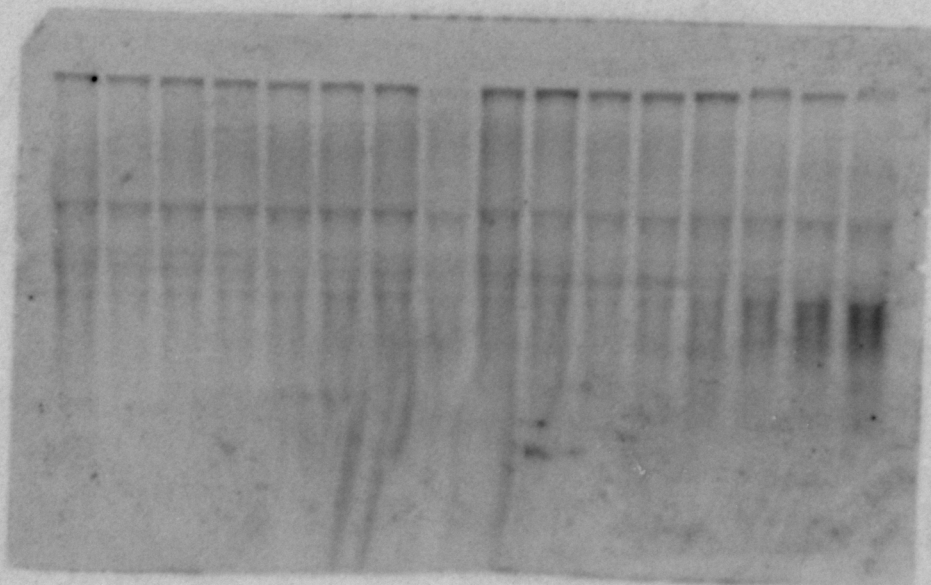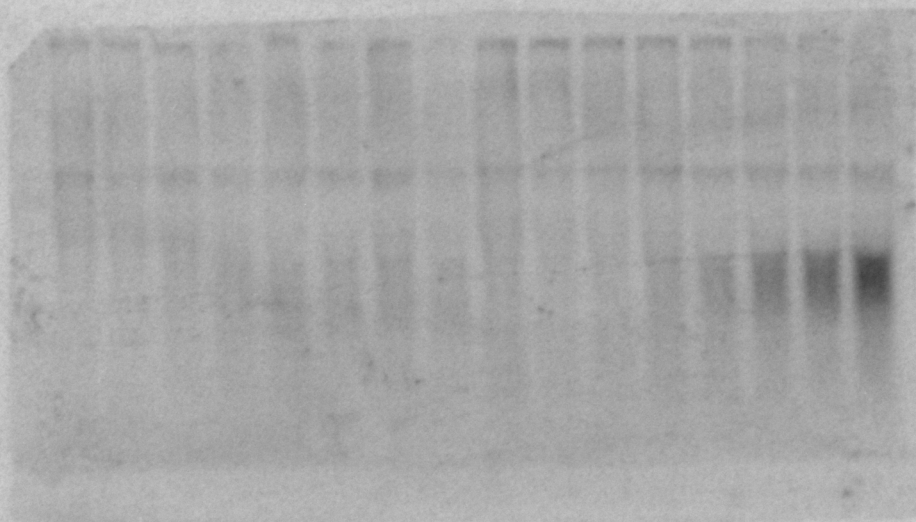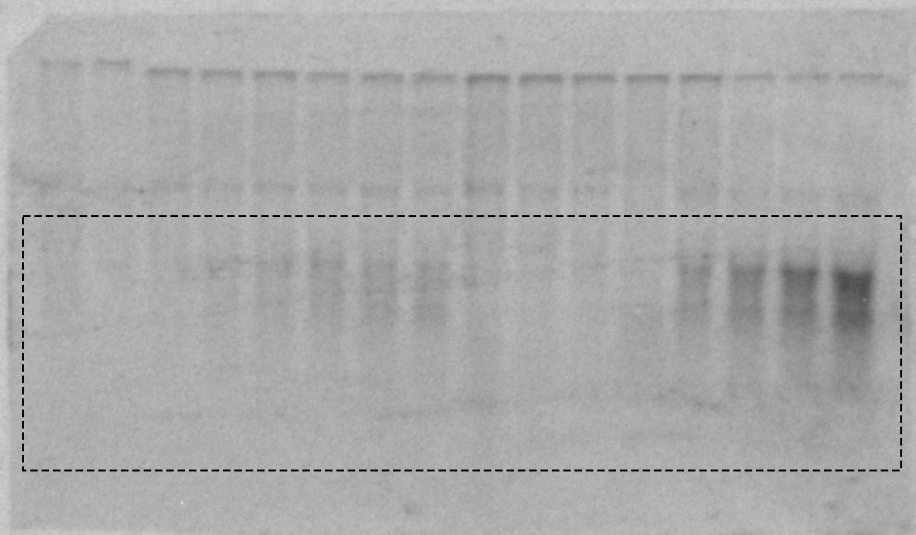

Supplement: Supplementary file 3 — Source data Fig. 1 [file 44319_2025_568_MOESM3_ESM.zip › Figure 1/1B Northern blot/SVN northern blot.pdf]

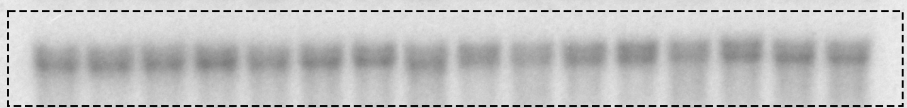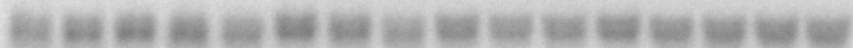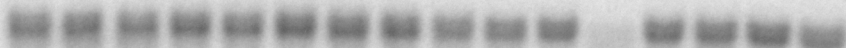

Supplement: Supplementary file 3 — Source data Fig. 1 [file 44319_2025_568_MOESM3_ESM.zip › Figure 1/1B Northern blot/UBI Northern blot.pdf]

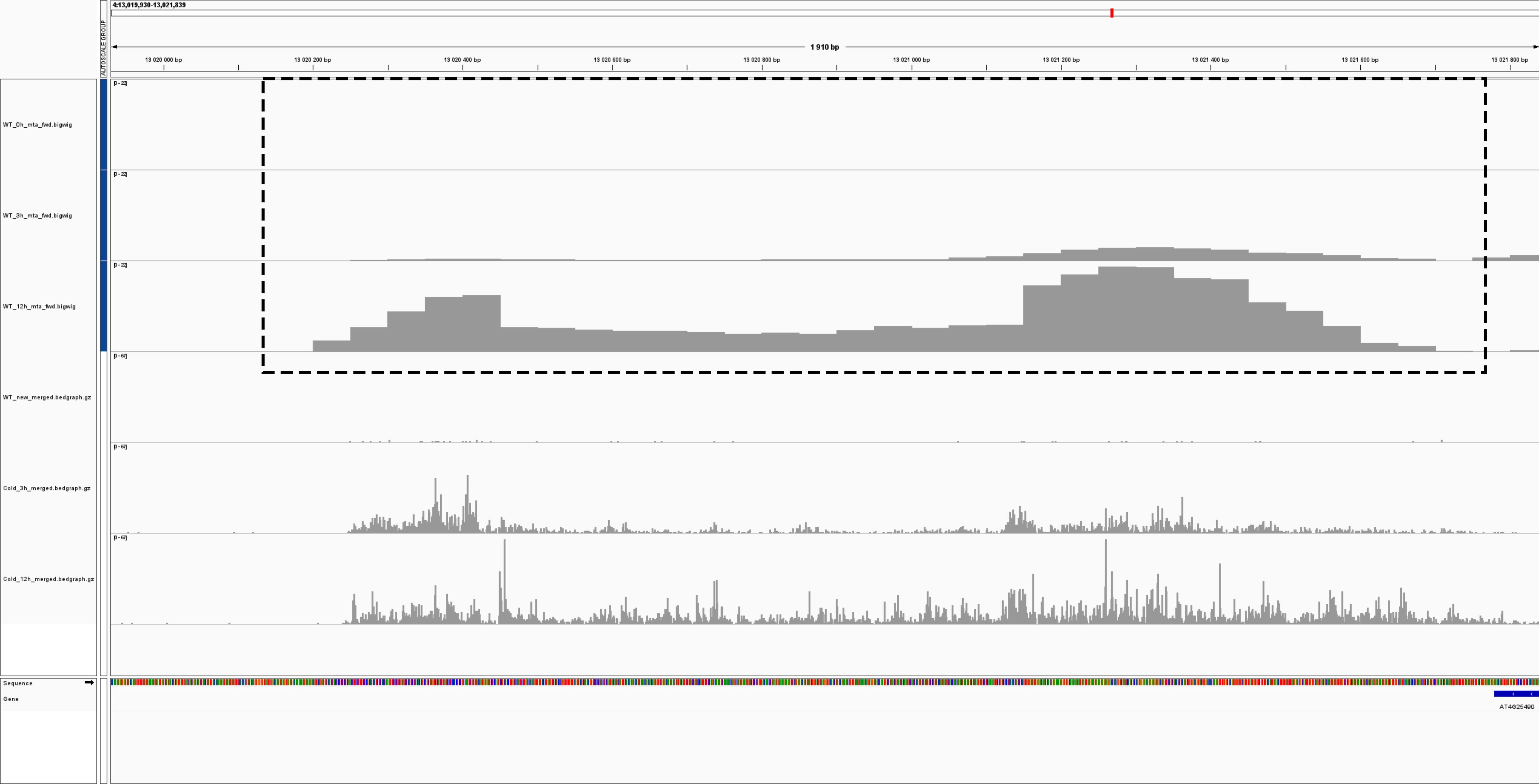

Supplement: Supplementary file 4 — Source data Fig. 2 [file 44319_2025_568_MOESM4_ESM.zip › Figure 2/2B Screenshot/SVK RNAseq screenshot.pdf]

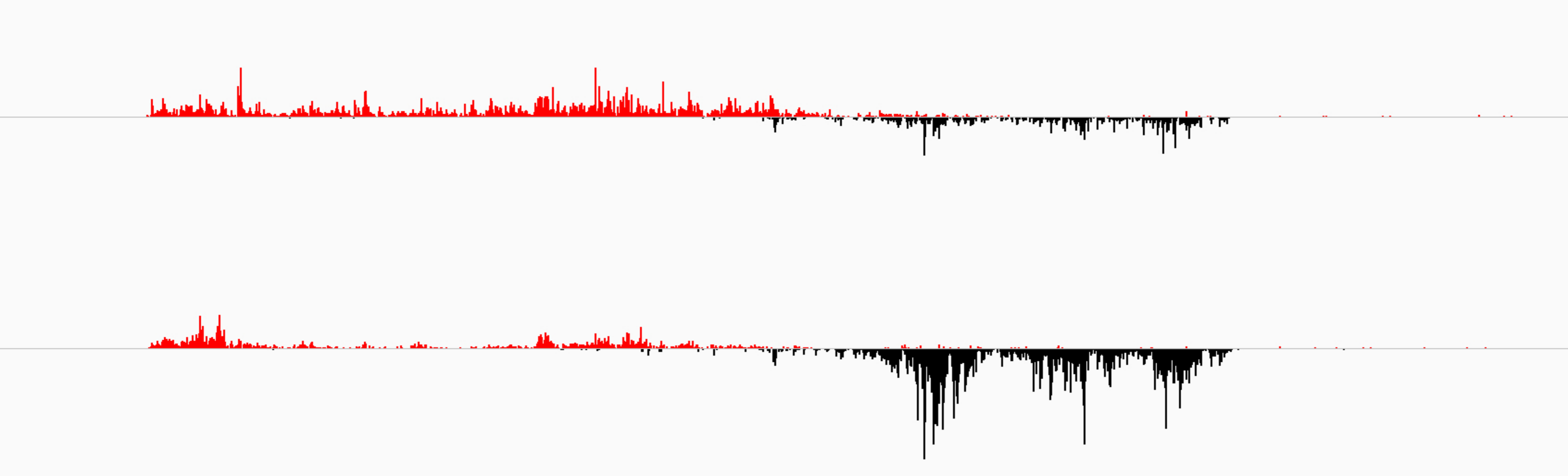

Supplement: Supplementary file 8 — Source data Fig. 6 [file 44319_2025_568_MOESM8_ESM.zip › Figure 6/6A screenshot/CBF1 SVK 3 and 12h cold.pdf]
