# Supplementary material for: Cis- and trans-action of the cold-induced lncRNAs, SVALKA and SVALNA, regulate CBF1 and CBF3 in Arabidopsis
Source: EMBO Rep. 2025 Sep 1;26(20):5070–87. doi: 10.1038/s44319-025-00568-5 (PMC12549994; doi:10.1038/s44319-025-00568-5)
Supplement: Supplementary file 10 — Expanded View Figures [file 44319_2025_568_MOESM10_ESM.pdf]

Expanded View Figures

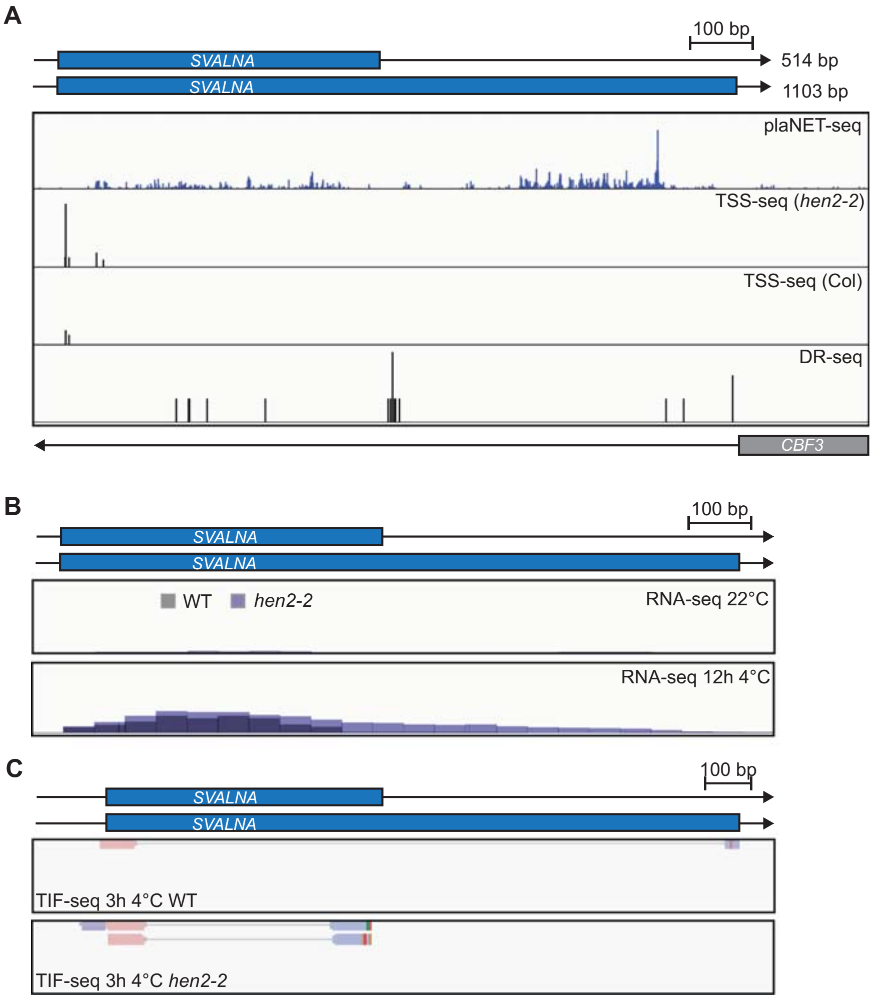

Figure EV1. Detection and characterization of *SVN* transcripts.

(**A**) plaNET-seq coverage profile for *SVN* with positions of RNAPII are shown for the sense strand in blue. TSS-seq (transcription start site sequencing) for WT and *hen2-2* and DR-seq (direct RNA sequencing) for WT tracks are also shown. The DR-seq track reveals sites of mRNA cleavage and polyadenylation (PAS). (**B**) Screenshot of the *SVN* locus from an RNA-seq dataset. Shown are WT and *hen2-2* at 22 °C and after 12 h at 4 °C. Elevated transcriptional activity is indicated by higher peak density and amplitude. (**C**) Screenshot of the *SVN* locus from a TIF-seq dataset. Shown are WT and *hen2-2* after 3 h at 4 °C.

© The Author(s)

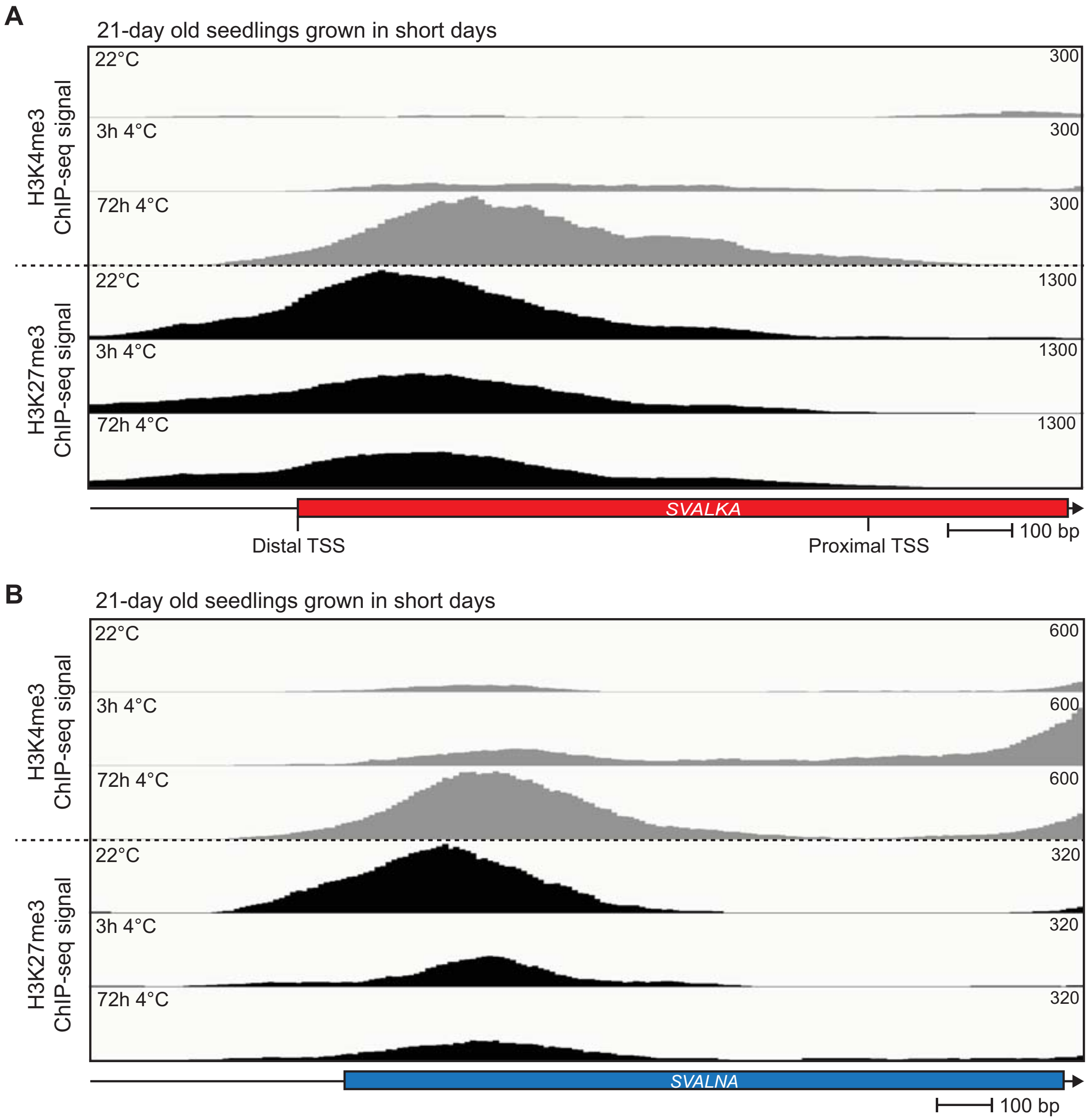

Figure EV2. Histone marks along the gene body of *SVK* and *SVN*.

(**A**) Screenshot of the *SVK* locus from a ChIP-seq dataset. Shown are WT at 22 °C and after 3 h and 72 h at 4 °C. Higher occupancy of H3K4me3 and H3K27me3 are indicated by higher peak density and amplitude. (**B**) Screenshot of the *SVN* locus from a ChIP-seq dataset. Shown are WT at 22 °C and after 3 h and 72 h at 4 °C. Higher occupancy of H3K4me3 and H3K27me3 are indicated by higher peak density and amplitude.

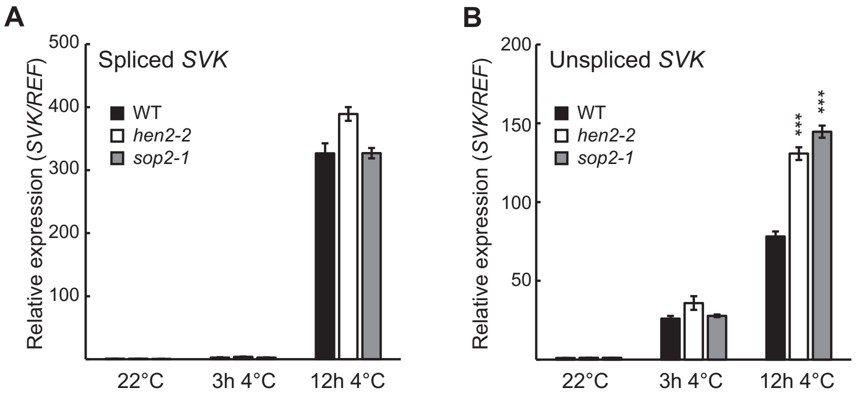

Figure EV3. Nuclear exosome sensitivity of spliced and unspliced *SVK* β.

(**A**, **B**) Relative spliced (**A**) and unspliced (**B**) *SVK* expression determined by RT-qPCR in WT, *hen2-2* and *sop2-1* mutants during cold conditions. Bars represent mean ± SEM from three biological replicates. The relative levels of transcripts were normalized to the level in WT in control conditions. Statistically significant differences between means were calculated with Student's *t* test (***$P < 0.001$). Exact *P* value for *hen2-2* was $P = 0.0023$. For *sop2-1*, exact *P* value was $P = 0.00082$.

 © The Author(s)

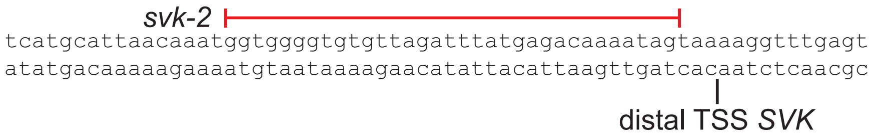

Figure EV4. Characterization of *SVK* CRISPR-Cas9 deletion line *svk-2.*

Graphical representation of the position of the deletion in the lines *svk-2* induced by CRIPSR-Cas9 used in this study.

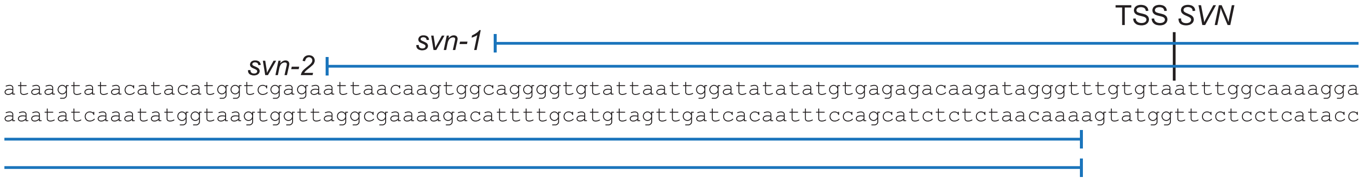

Figure EV5. Characterization of *SVN* CRISPR-Cas9 deletion lines *svn-1* and *svn-2*.

Graphical representation of the position of the deletion in the lines *svn-1* and *svn-2* induced by CRIPSR-Cas9 used in this study.

© The Author(s)
