# Supplementary material for: Cis- and trans-action of the cold-induced lncRNAs, SVALKA and SVALNA, regulate CBF1 and CBF3 in Arabidopsis
Source: EMBO Rep. 2025 Sep 1;26(20):5070–87. doi: 10.1038/s44319-025-00568-5 (PMC12549994; doi:10.1038/s44319-025-00568-5)
Supplement: Supplementary file 2 — Peer Review File [file 44319_2025_568_MOESM2_ESM.pdf]

EMBO reports

Cis- and trans-action of the cold-induced lncRNAs, SVALKA and SVALNA, regulate CBF1 and CBF3

Peter Kindgren, Isabell Rosenkranz, Sarah Mermet, and Vasiliki Zacharaki

Corresponding author(s): Peter Kindgren (peter.kindgren@slu.se)

Review Timeline:

Submission Date:	15th May 25
Editorial Decision:	9th Jun 25
Revision Received:	26th Jul 25
Editorial Decision:	15th Aug 25
Revision Received:	18th Aug 25
Accepted:	20th Aug 25

Editor: Yehu Moran

Transaction Report:

(Note: With the exception of the correction of typographical or spelling errors that could be a source of ambiguity, letters and reports are not edited. Depending on transfer agreements, referee reports obtained elsewhere may or may not be included in this compilation. Referee reports are anonymous unless the Referee chooses to sign their reports.)

1st Editorial Decision 9th Jun 2025

Dear Dr. Kindgren

Thank you for the submission of your manuscript to EMBO Reports. We have now received the full set of referee reports as well as referee cross-comments that are all pasted below.

As you will see, all three referees find your results interesting. However, they do raise various comments and concerns that require your attention. Furthermore, Referee #2 suggests a major change in the order of the results as they believe this can make the story more appealing to the readers. I encourage you to explore this suggestion as it sounds to me quite promising. Yet, if you eventually choose not to follow it I ask you to provide a detailed explanation why you believe the current structure is better than the suggested one.

I would thus like to invite you to revise your manuscript with the understanding that all the referee concerns must be fully addressed and their suggestions taken on board. Please address all referee concerns in a complete point-by-point response. Acceptance of the manuscript will depend on a positive outcome of a second round of review. It is EMBO Reports policy to allow a single round of major revision only and acceptance or rejection of the manuscript will therefore depend on the completeness of your responses included in the next, final version of the manuscript.

We realize that it is difficult to revise to a specific deadline. In the interest of protecting the conceptual advance provided by the work, we recommend a revision within 3 months (9th Sep 2025). Please discuss the revision progress ahead of this time with the editor if you require more time to complete the revisions.

You can either publish the study as a short report or as a full article. For short reports, the revised manuscript should not exceed 27,000 characters (including spaces but excluding materials & methods and references) and 5 main plus 5 expanded view figures. The results and discussion sections must further be combined, which will help to shorten the manuscript text by eliminating some redundancy that is inevitable when discussing the same experiments twice. For a normal article there are no length limitations, but it should have more than 5 main figures and the results and discussion sections must be separate. In both cases, the entire materials and methods must be included in the main manuscript file.

IMPORTANT NOTE: we perform an initial quality control of all revised manuscripts before re-review. Your manuscript will FAIL this control and the handling will be DELAYED if the following APPLIES:
1) A data availability section providing access to data deposited in public databases is missing. If you have not deposited any data, please add a sentence to the data availability section that explains that.
2) Your manuscript contains statistics and error bars based on n=2. Please use scatter blots in these cases. No statistics should be calculated if n=2.

When submitting your revised manuscript, please carefully review the instructions that follow below. Failure to include requested items will delay the evaluation of your revision.

1) a .docx formatted version of the manuscript text (including legends for main figures, EV figures and tables). Please make sure that the changes are highlighted to be clearly visible.

2) individual production quality figure files as .eps, .tif, .jpg (one file per figure). See https://wol-prod-cdn.literatumonline.com/pb-assets/embo-site/EMBOPress_Figure_Guidelines_061115-1561436025777.pdf for more info on how to prepare your figures.

3) We replaced Supplementary Information with Expanded View (EV) Figures and Tables that are collapsible/expandable online. A maximum of 5 EV Figures can be typeset. EV Figures should be cited as 'Figure EV1, Figure EV2" etc... in the text and their respective legends should be included in the main text after the legends of regular figures.

- For the figures that you do NOT wish to display as Expanded View figures, they should be bundled together with their legends in a single PDF file called *Appendix*, which should start with a short Table of Content. Appendix figures should be referred to in the main text as: "Appendix Figure S1, Appendix Figure S2" etc. See detailed instructions regarding expanded view here: <https://www.embopress.org/page/journal/14693178/authorguide#expandedview>

- Additional Tables/Datasets should be labeled and referred to as Table EV1, Dataset EV1, etc. Legends have to be provided in a separate tab in case of .xls files. Alternatively, the legend can be supplied as a separate text file (README) and zipped together with the Table/Dataset file.

4) a .docx formatted letter INCLUDING the reviewers' reports and your detailed point-by-point responses to their comments. As part of the EMBO Press transparent editorial process, the point-by-point response is part of the Review Process File (RPF), which will be published alongside your paper.

5) a complete author checklist, which you can download from our author guidelines

<https://www.embopress.org/page/journal/14693178/authorguide>. Please insert information in the checklist that is also reflected in the manuscript. The completed author checklist will also be part of the RPF.

6) Please note that all corresponding authors are required to supply an ORCID ID for their name upon submission of a revised manuscript (<https://orcid.org/>). Please find instructions on how to link your ORCID ID to your account in our manuscript tracking system in our Author guidelines
<https://www.embopress.org/page/journal/14693178/authorguide#authorshipguidelines>

7) Before submitting your revision, primary datasets produced in this study need to be deposited in an appropriate public database (see https://www.embopress.org/page/journal/14693178/authorguide#datadeposition). Please remember to provide a reviewer password if the datasets are not yet public. The accession numbers and database should be listed in a formal "Data Availability" section placed after Materials & Method (see also https://www.embopress.org/page/journal/14693178/authorguide#datadeposition). Please note that the Data Availability Section is restricted to new primary data that are part of this study. * Note - All links should resolve to a page where the data can be accessed. *
If your study has not produced novel datasets, please mention this fact in the Data Availability Section.

8) At EMBO Press we ask authors to provide source data for the main manuscript figures. You will receive a separate email with instructions for providing source data with your revised manuscript, including how to upload and organize the files.

9) Our journal also encourages inclusion of *data citations in the reference list* to directly cite datasets that were re-used and obtained from public databases. Data citations in the article text are distinct from normal bibliographical citations and should directly link to the database records from which the data can be accessed. In the main text, data citations are formatted as follows: "Data ref: Smith et al, 2001" or "Data ref: NCBI Sequence Read Archive PRJNA342805, 2017". In the Reference list, data citations must be labeled with "[DATASET]". A data reference must provide the database name, accession number/identifiers and a resolvable link to the landing page from which the data can be accessed at the end of the reference. Further instructions are available at https://www.embopress.org/page/journal/14693178/authorguide#referencesformat

10) Regarding data quantification (see Figure Legends:
https://www.embopress.org/page/journal/14693178/authorguide#figureformat)

The following points must be specified in each figure legend:

- the name of the statistical test used to generate error bars and P values,

- the number (n) of independent experiments (please specify technical or biological replicates) underlying each data point,

- the nature of the bars and error bars (s.d., s.e.m.),

- If the data are obtained from n Program fragment delivered error ``Can't locate object method "less" via package "than" (perhaps you forgot to load "than"?) at //ejpvfs23/sites23b/embor_www/letters/embor_decision_revise_and_review.txt line 56.' 2, use scatter blots showing the individual data points.

Discussion of statistical methodology can be reported in the materials and methods section, but figure legends should contain a basic description of n, P and the test applied.

- Please also include scale bars in all microscopy images.

11) The journal requires a statement specifying whether or not authors have competing interests (defined as all potential or actual interests that could be perceived to influence the presentation or interpretation of an article). In case of competing interests, this must be specified in your disclosure statement. Further information: https://www.embopress.org/competing-interests

12) All Materials and Methods need to be described in the main text using our 'Structured Methods' format, which is required for all research articles. According to this format, the Methods section includes a Reagents and Tools Table (listing key reagents, experimental models, software and relevant equipment and including their sources and relevant identifiers) followed by a Methods and Protocols section describing the methods using a step-by-step protocol format. The aim is to facilitate adoption of the methodologies across labs. More information on how to adhere to this format as well as a downloadable template (.docx) for the Reagents and Tools Table can be found in our author guidelines:
https://www.embopress.org/page/journal/14693178/authorguide#structuredmethods.

An example of a Method paper with Structured Methods can be found here: https://www.embopress.org/doi/full/10.1038/s44320-024-00037-6#sec-4

We would also welcome the submission of cover suggestions, or motifs to be used by our Graphics Illustrator in designing a cover.

As part of the EMBO publication's Transparent Editorial Process, EMBO reports publishes online a Review Process File (RPF) to accompany accepted manuscripts. This File will be published in conjunction with your paper and will include the referee reports, your point-by-point response and all pertinent correspondence relating to the manuscript.

You are able to opt out of this by letting the editorial office know (emboreports@embo.org). If you do opt out, the Review Process File link will point to the following statement: "No Review Process File is available with this article, as the authors have chosen not to make the review process public in this case."

I look forward to seeing a revised form of your manuscript when it is ready.

Yours sincerely,

Yehu Moran
Academic Editor
EMBO Reports

Referee #1:

The manuscript presents an analysis of SVALKA and SVALNA transcriptional regulation of CBF1 and CBF3 genes. The use of Arabidopsis mutants and design of qRTPCR primers enables identification of different SVALKA isoforms at different stages of response to temperature stress. A CRISPR Cas9 deletion strategy generated mutants at the promoter site for SVN. More detail in the methods, a diagram of the vector, and a reference citation about the CF588 vector would be helpful in understanding how the mutant was generated. The authors make good use of publicly available sequencing data sets. Including the sequence repository database and identifiers in addition to the literature citation (line 333) would facilitate other researcher's use of these data. As a minor point, an acknowledgement and citation of the COOLAIR secondary structure by in vivo chemical probing (Nature volume 609, pages394-399 (2022)) could be added to line 246. The results are exciting and worth publishing with minor revisions.

Referee #2:

In this work, Rosenkranz and colleagues study the role of 2 lncRNAs, one acting in cis and in trans, while the second one acts primarily in cis due to its intrinsecal stability. The Kindgren lab has already published very compelling data about the first of these lncRNAs, SVALKA, and now introduces SVALNA (overlapped to CBF3, a gene regulated in trans by SVALKA).
This work offers very interesting data, which is very valuable for the plant noncoding RNA comunity. However, to be honest, I find the results organized in a way that doesn't allow reflecting the conclusions in order to deliver a clear and straight-forward take-away message.
I will suggest an alternative way to tell the story, which as an interested reader, I think it'd be more profitable for me... I hope it helps!

First of all, the title of the article:"via multiple mechanisms" sounds too vague. I suggest focusing on the main finding (here's an idea to start with): "Cis and trans action of cold-induced lncRNAs regulate the expression of COLD BINDING FACTORS genes in Arabidopsis"
Second, the first section of the Results should be integrated into another section. It's sad to start a new paper in a major jounral devoting a section to a "confirmation"! It sounds as incremental work, wheareas here the authors identify a novel lncRNA contributing to the regulation of a TF gene. I'd definetly start by this identification...
Then, I suggest organizing the finding comparing step by step both lncRNAs (in this or another order):

1. epigenetic regulation of SVK and SVN. Compare H3K27m3 and H3K4m3 over both lncRNA loci in parallel.
2. splicing regulation of BOTH (SVN is not subjected to alternative splicing?). Highlight differences between SVN and SVK.
3. exosome-mediated degradation of both lncRNAs. A kinetics in response to cold to assess SVK, SVN, CBF1 and 3 over time would be a nice plus.
4. genetics over both loci to uncover cis and potential cross-trans actions of both lncRNAs (notably positive for SVK and negative for SVN).

Overall, I strongly suggest focusing on the fact that SVN is rapidly degraded, thus it only acts in cis, whereas specific isoforms of SVK are more stable and can regulate another locus in trans. And that all this happens as successive steps during long exposure to cold.

Some additional suggestions:
in the abstract, the phrase "WE used Native Elongation Transcript Sequencing, CRISPR-Cas9 deletion strategies, and RT-qPCR to analyze" would be better as "We integrated omics approaches, together with genetics and molecular biology to uncover..."

Also in the abstract, the authors mention that specific isoforms of SVK are more stable thanks to certain structures. This is not studied in the manuscrtipt at all, only integrated in the Discussion. This should be toned down.

In the Discussion, it'd be nice to inegrate literature about plant lncRNAs in response to cold (APOLO, patrticularly in Moison et al 2021), VAS in wheat, in addition to those already discussed. It'd be awesome to discuss what is going on with noncoding transcription in response to cold. Are lncRNAs thermosensors? What do the author think about this issue?

I hope my feedback helps.

Referee #3:

The authors report an investigation of the lncRNA SVALKA (SVK) and the identification of a novel lncRNA involved in regulating gene expression under cold stress conditions. They named this newly identified lncRNA SVALNA (SVN). They corroborate and further characterise the function of SVALKA through the use of insertional mutants as well as the generation of CRISPR deletions in both the promoter of SVALNA and the Distal isoform of SVALKA. Overall, the identification of SVALKA raises the question of whether an antisense collision mechanism might be more widespread in regulating the cold response in Arabidopsis and other plants.

However, I have several comments regarding the manuscript's findings:

Major comments

1) The results overall seem rather solid for the first section, including the identification of SVN; however, when the authors discuss how SVN negatively regulates CBF3, there are several unclear sections. Specifically, publicly available ChIP-seq datasets used are not adequately described. It is mentioned that these are for repressive and activating marks, but there is no description of the conditions under which these are used. I had to refer back to the original paper, Feivre et al. (2024), to find that the data were acquired from 21-day-old seedlings treated at 4 {degree sign}C. This information is not mentioned in the text, methods, or figures. Although they are not a precise match, given that the authors used 10-day-old seedlings, having this information will allow readers to consider the findings in light of the experimental differences. Following up on this, if the conditions were to be matched, the authors could extend their discussion to suggest that SVALNA might be triggering a chromatin change.

2) In Line 141, the authors state that the SVALNA switches poly(A) site during prolonged cold exposure, in a manner similar to SVALKA. However, no clear evidence for this is provided in the manuscript. The transcript and RNA-Seq tracks shown in Supp Fig 1 do not convince me that these are the two isoforms of these transcripts. Can the two isoforms be assembled from the RNA-seq data? Is there any long-read data available that supports the two isoforms shown on Supp Fig 1? This can be derived from in-house or public datasets; however, more evidence is needed to support the purported two isoforms of SVALNA and to substantiate the claim of switching poly(A) sites, particularly given that SVALNA expression is noticeably lower (Figure 2A) than that of SVALKA (Figure 1A).

3) In Line 161, the authors discuss how the accumulation of SVALKA transcript indicates that most amplicons represent unspliced forms of SVK beta. This statement is difficult to follow, as the text refers to shorter transcripts (SVK alpha) and transcripts from a Distal TSS as SVK beta. However, in Figure 4, there is no legend mentioning which one is which. I had to refer to Figure 1 of Kindgren et al. (2018) to understand what they meant, as I originally interpreted that the isoform at the top of Figure 4A was SVK alpha and the bottom SVK beta, which is not the case. This should be made clearer, ideally by making the diagram as detailed as Figure 1d of Kindgren et al. (2018).

4) Furthermore, in line 154 & Figure 4, the authors claim to use qPCR probes that, in theory, allow them to differentiate between isoforms. Indeed, the Distal probe should allow them to measure the expression of Distal isoforms (SVK beta). However, the Proximal qPCR probe overlaps both isoforms, yet the results do not correspond to what was found for the Distal isoform. The authors must provide more information on how the Proximal probe was designed to capture only the Proximal isoforms (SVK alpha). Please revise this section and the figure to clarify how this probe differentiates between isoforms. If it does not, it would be appropriate to corroborate these findings with a different set of qPCR probes.

5) The colour scheme of the figures describing RT-PCR results (particularly Figures 1, 2, 3, and 5) is aesthetically pleasing and seems to be the author's preferred style, based on reading other previous publications. However, using different shades of the same colour makes it difficult to rapidly identify the corresponding results. This may be even more challenging for someone with colour blindness. I strongly recommend considering using different colours that enhance rapid understanding of RT-PCR results, or adding the names below each column to enhance understanding. I found the black and white pictures easier to grasp quickly.

It is worth sacrificing aesthetics for the sake of message clarity.

6) In the methods section (starting on line 333), more details should be provided on how the public datasets were used - were they reanalysed? Were only bigWigs downloaded from NCBI's GEO? I'm all for reusing public data, but it should be clear how it was analyzed and how that impacts the findings.

Minor comments

The grammar and language can be improved in several instances:

- Line 78 - The authors write ".. temporal regulation of distinct isoforms of lncRNAs to achieve regulatory flexibility in response to unfavourable conditions". I suggest changing the end to "changing environmental conditions" for clarity and also to reduce the load of having cold be unfavourable, when it is a naturally occurring environmental variation linked to the changing seasons.
- Line 137 - "data set" should be "dataset"
- Line 148 - Change "earlier" to "previously" for clarity
- Line 186 - Change "marks at control conditions" to "marks in control conditions"
- Line 281 - Soften statement " they are the cornerstone of complex gene regulation" to "they are one of the cornerstones of complex gene regulation", as there are many other regulators besides lncRNAs, as fascinating as they are.

Response to reviewers EMBOR-2025-61946-T

We would like to thank the three anonymous referees and the editor for their insights and constructive feedback. We have compiled all concerns and our responses in this document. Comments are in black and our response in red text.

Referee #1:

The manuscript presents an analysis of SVALKA and SVALNA transcriptional regulation of CBF1 and CBF3 genes. The use of Arabidopsis mutants and design of qRTPCR primers enables identification of different SVALKA isoforms at different stages of response to temperature stress. A CRISPR Cas9 deletion strategy generated mutants at the promoter site for SVN. More detail in the methods, a diagram of the vector, and a reference citation about the CF588 vector would be helpful in understanding how the mutant was generated. The authors make good use of publicly available sequencing data sets. Including the sequence repository database and identifiers in addition to the literature citation (line 333) would facilitate other researcher's use of these data. As a minor point, an acknowledgement and citation of the COOLAIR secondary structure by in vivo chemical probing (Nature volume 609, pages394-399 (2022)) could be added to line 246. The results are exciting and worth publishing with minor revisions.

We thank the referee for the positive review. We revised the method section and added a reference for the original binary vector (pHSE401) from which the CF588 vector derives. We also added identifiers to the datasets that were used in this study and how they were analyzed. Additionally, we also followed your advice and cited Yang et al, 2022 (COOLAIR secondary structure by in vivo chemical probing).

Referee #2:

In this work, Rosenkranz and colleagues study the role of 2 lncRNAs, one acting in cis and in trans, while the second one acts primarily in cis due to its intrinsecal stability. The Kindgren lab has already published very compelling data about the first of these lncRNAs, SVALKA, and now introduces SVALNA (overlapped to CBF3, a gene regulated in trans by SVALKA). This work offers very interesting data, which is very valuable for the plant noncoding RNA community. However, to be honest, I find the results organized in a way that doesn't allow reflecting the conclusions in order to deliver a clear and straight-forward take-away message. I will suggest an alternative way to tell the story, which as an interested reader, I think it'd be more profitable for me... I hope it helps!

First of all, the title of the article:"via multiple mechanisms" sounds too vague. I suggest focusing on the main finding (here's an idea to start with): "Cis and trans action of cold-induced lncRNAs regulate the expression of COLD BINDING FACTORS genes in Arabidopsis"
Second, the first section of the Results should be integrated into another section. It's sad to start a new paper in a major jounral devoting a section to a "confirmation"! It sounds as

incremental work, wheareas here the authors identify a novel lncRNA contributing to the regulation of a TF gene. I'd definetly start by this identification...

Thank you for your insights and recommendations. We have changed the title to *"Cis- and trans-action of the cold-induced long non-coding RNAs, SVALKA and SVALNA, regulate CBF1 and CBF3 in Arabidopsis."* We agree that this better captures the main message of the manuscript. In addition, we have followed your suggestion and started the results with the identification of *SVALNA*.

Then, I suggest organizing the finding comparing step by step both lncRNAs (in this or another order):
1. epigenetic regulation of SVK and SVN. Compare H3K27m3 and H3K4m3 over both lncRNA loci in parallel.

2. splicing regulation of BOTH (SVN is not subjected to alternative splicing?). Highlight differences between SVN and SVK.

3. exosome-mediated degradation of both lncRNAs. A kinetics in response to cold to assess SVK, SVN, CBF1 and 3 over time would be a nice plus.

4. genetics over both loci to uncover cis and potential cross-trans actions of both lncRNAs (notably positive for SVK and negative for SVN).

Overall, I strongly suggest focusing on the fact that SVN is rapidly degraded, thus it only acts in cis, whereas specific isoforms of SVK are more stable and can regulate another locus in trans. And that all this happens as successive steps during long exposure to cold.

Thank you for these suggestions and we agree that comparing the two lncRNAs throughout the results might result in a more compelling story. In our resubmitted manuscript, we start the results with the identification of *SVN* (Fig. 1) and the identification of the different isoforms of *SVK* (Fig. 2). The epigenetic (Supp. Fig. 2), exosome sensitivity (Fig. 3), splicing (Fig. 4) and genetics (Fig. 6-7) results are now presented together to highlight the similarities and differences between *SVK* and *SVN*. The text has also been updated to highlight these characteristics of the lncRNAs better.

Some additional suggestions:

In the abstract, the phrase "We used Native Elongation Transcript Sequencing, CRISPR-Cas9 deletion strategies, and RT-qPCR to analyze" would be better as "We integrated omics approaches, together with genetics and molecular biology to uncover..."

We have changed the sentence as suggested.

Also in the abstract, the authors mention that specific isoforms of SVK are more stable thanks to certain structures. This is not studied in the manuscript at all, only integrated in the Discussion. This should be toned down.

Agreed, we have toned this point in the abstract.

In the Discussion, it'd be nice to integrate literature about plant lncRNAs in response to cold (APOLO, particularly in Moison et al 2021), VAS in wheat, in addition to those already discussed. It'd be awesome to discuss what is going on with noncoding transcription in response to cold. Are lncRNAs thermosensors? What do the author think about this issue?

I hope my feedback helps.

Thank you for the suggested additions. We followed your advice and included *APOLO* and *VAS* in the discussion. We also discussed lncRNAs as possible thermosensors. As we point out in the added text, there is no evidence in plants or other eukaryotes that lncRNAs can work as thermosensors yet. It is, however, an exciting prospect worth investigating in the future.

Referee #3:

The authors report an investigation of the lncRNA SVALKA (SVK) and the identification of a novel lncRNA involved in regulating gene expression under cold stress conditions. They named this newly identified lncRNA SVALNA (SVN). They corroborate and further characterise the function of SVALKA through the use of insertional mutants as well as the generation of CRISPR deletions in both the promoter of SVALNA and the Distal isoform of SVALKA. Overall, the identification of SVALKA raises the question of whether an antisense collision mechanism might be more widespread in regulating the cold response in Arabidopsis and other plants.

However, I have several comments regarding the manuscript's findings:

Major comments

1) The results overall seem rather solid for the first section, including the identification of SVN; however, when the authors discuss how SVN negatively regulates CBF3, there are several unclear sections. Specifically, publicly available ChIP-seq datasets used are not adequately described. It is mentioned that these are for repressive and activating marks, but there is no description of the conditions under which these are used. I had to refer back to the original paper, Feivre et al. (2024), to find that the data were acquired from 21-day-old seedlings treated at 4 {degree sign}C. This information is not mentioned in the text, methods, or figures. Although they are not a precise match, given that the authors used 10-day-old seedlings, having this information will allow readers to consider the findings in light of the experimental differences. Following up on this, if the conditions were to be matched, the authors could extend their discussion to suggest that SVALNA might be triggering a chromatin change.

Thank you for pointing this out. We have added information about the experimental design from Feivre et al in the results. We have also added more text in Supp. Fig. 2, so it is easier for the reader to consider the differences in experimental design. That said, we have done multiple experiments in the lab with long day and short day grown seedlings at different ages and see similar *CBF* and *SVK* expression patterns in response to 4°C. H3K4me3 usually

follows transcriptional activity well and the H3K4me3 pattern we detect in the ChIP-seq data reflects our RT-qPCR results. It is therefore unlikely that there are large discrepancies between the samples in terms of *CBF* and *SVK/SVN* chromatin changes. However, there are currently no ChIP-seq dataset that replicate our conditions, so we have refrained from drawing any further conclusions in this part of the manuscript.

2) In Line 141, the authors state that the SVALNA switches poly(A) site during prolonged cold exposure, in a manner similar to SVALKA. However, no clear evidence for this is provided in the manuscript. The transcript and RNA-Seq tracks shown in Supp Fig 1 do not convince me that these are the two isoforms of these transcripts. Can the two isoforms be assembled from the RNA-seq data? Is there any long-read data available that supports the two isoforms shown on Supp Fig 1? This can be derived from in-house or public datasets; however, more evidence is needed to support the purported two isoforms of SVALNA and to substantiate the claim of switching poly(A) sites, particularly given that SVALNA expression is noticeably lower (Figure 2A) than that of SVALKA (Figure 1A).

Thank you for this comment. To address this, we have added Transcript Isoform sequencing (TIF-seq) data from Thomas et al (2020) to Supp. Fig. 1C. TIF-seq sequence start and ends of transcripts and we find support for both *SVN* isoforms at 4°C. We clearly show that *SVN* has a preferred start site and two end sites (poly(A)-signals) in Supp. Fig. 1 that corresponds to the isoforms we detect with Northern blot. We do not claim that *SVN* switches poly(A)-signal during cold treatment, merely stating that the longer isoform is more prevalent later in the cold response (as seen on the Northern blot and supported by higher transcriptional activity in the plaNET-seq data). We have also added SVN isoform RT-qPCR data to Fig. 1 that supports the other data points.

3) In Line 161, the authors discuss how the accumulation of SVALKA transcript indicates that most amplicons represent unspliced forms of SVK beta. This statement is difficult to follow, as the text refers to shorter transcripts (SVK alpha) and transcripts from a Distal TSS as SVK beta. However, in Figure 4, there is no legend mentioning which one is which. I had to refer to Figure 1 of Kindgren et al. (2018) to understand what they meant, as I originally interpreted that the isoform at the top of Figure 4A was SVK alpha and the bottom SVK beta, which is not the case. This should be made clearer, ideally by making the diagram as detailed as Figure 1d of Kindgren et al. (2018).

This is a good point, and we have tried to address it in the resubmitted manuscript. In the updated Fig. 2C, we have included all *SVK* isoforms, and the RT-qPCR probes used to amplify them. We have also pointed out which isoforms is defined as *SVK* α and *SVK* β in the figure.

4) Furthermore, in line 154 & Figure 4, the authors claim to use qPCR probes that, in theory, allow them to differentiate between isoforms. Indeed, the Distal probe should allow them to measure the expression of Distal isoforms (SVK beta). However, the Proximal qPCR probe overlaps both isoforms, yet the results do not correspond to what was found for the Distal isoform. The authors must provide more information on how the Proximal probe was designed to capture only the Proximal isoforms (SVK alpha). Please revise this section and

the figure to clarify how this probe differentiates between isoforms. If it does not, it would be appropriate to corroborate these findings with a different set of qPCR probes.

We agree that this was not adequately addressed in the first submission. To address this concern, we have made several changes to the manuscript. Firstly, we have included all RT-qPCR probes for SVK in the updated Fig. 2 and included spliced and unspliced *SVK β* expression levels for WT in the figure. Secondly, we have included RT-qPCR data for spliced and unspliced *SVK β* in the exosome mutants *hen2-2* and *sop2-1* in Sup. Fig. 3. With these new data points, it is clear that unspliced *SVK β* is targeted by the nuclear exosome. The proximal *SVK* (*SVK α*) is not an exosome target at 3h 4°C (Fig. 3C), but at 12h when unspliced *SVK β* accumulates. It is challenging to only amplify one of the transcripts but by extrapolating the results from the proximal probe and unspliced *SVK β,* we believe that it is possible to draw the conclusion stated in the manuscript.

5) The colour scheme of the figures describing RT-PCR results (particularly Figures 1, 2, 3, and 5) is aesthetically pleasing and seems to be the author's preferred style, based on reading other previous publications. However, using different shades of the same colour makes it difficult to rapidly identify the corresponding results. This may be even more challenging for someone with colour blindness. I strongly recommend considering using different colours that enhance rapid understanding of RT-PCR results or adding the names below each column to enhance understanding. I found the black and white pictures easier to grasp quickly. It is worth sacrificing aesthetics for the sake of message clarity.

Point taken, we have changed the colour scheme accordingly.

6) In the methods section (starting on line 333), more details should be provided on how the public datasets were used - were they reanalysed? Were only bigWigs downloaded from NCBI's GEO? I'm all for reusing public data, but it should be clear how it was analyzed and how that impacts the findings.

We fully agree with this important point and have tried to update the manuscript accordingly in the method section. Briefly, the RNA-seq, plaNET-seq, TSS-seq, and TIF-seq were all done in the lab and analysed according to the respective paper referred to in the methods. The ChIP-seq data was downloaded as bigwig files from the depository. The DRS-seq files were remapped according to Kindgren et al, 2020.

Minor comments

The grammar and language can be improved in several instances:

- Line 78 - The authors write ".. temporal regulation of distinct isoforms of lncRNAs to achieve regulatory flexibility in response to unfavourable conditions". I suggest changing the end to "changing environmental conditions" for clarity and also to reduce the load of having cold be unfavourable, when it is a naturally occurring environmental variation linked to the changing seasons.
- Line 137 - "data set" should be "dataset"

- Line 148 - Change "earlier" to "previously" for clarity

- Line 186 - Change "marks at control conditions" to "marks in control conditions"

- Line 281 - Soften statement " they are the cornerstone of complex gene regulation" to "they are one of the cornerstones of complex gene regulation", as there are many other regulators besides lncRNAs, as fascinating as they are.

We thank you for your language and grammar improvements. We have followed your advice on all points.

Dear Dr. Kindgren

Thank you for the submission of your revised manuscript to our offices. We have now received the enclosed reports from the referees that were asked to assess it. While all three reviewers were satisfied with the revision our editorial assistance team still has minor technical requirements that I would like you to incorporate before we can proceed with the official acceptance of your manuscript.

Our automatic deadline for revisions is 3 months, but I imagine you can send it your revised manuscript much faster than that in light of the very minor nature of our comments.

I look forward to seeing a new revised version of your manuscript.

Best regards,
Yehu Moran
Academic Editor
EMBO Reports

Data Accessibility Statement: needs to be placed before Acknowledgments
REFERENCES: et al needs to be used after the 10th author name (if a reference has more than 10 authors).
FUNDING INFO: grant number 2018-03926 is in the system, missing in the manuscript text; funding info needs to be congruent, please correct.
DATASET EV LEGENDS: Table EV1 has wrong title in the Excel - Expanded View Table 1. Please correct.
SYNOPSIS IMAGE: missing, please provide (see examples on our website).
SYNOPSIS TEXT: missing, please provide (see examples on our website).

NOTES:
- Methods and Material should be renames Methods

Figure Legends - Comments
- Please note that the exact p values are not provided in the legends of figures 3A-D; 4A-C; 6C, D; 7B, C; EV 3 B. Please provide.

REFEREE REPORTS:

Referee #1

The authors have addressed clearly all the points in the reviews. The information about the online data sets and the vector is much better. The discussion about thermosensors and thermoresponsive lncRNA is very interesting and enhances the manuscript.

Referee #2:

Congratulations to the authors for this valuable contribution to the field of plant lncRNAs. I'm looking forward to seeing this work published.
Federico Ariel

Referee #3:

I appreciate the authors carefully addressing each of the points I raised in my review. I'm satisfied with the changes and believe the revised manuscript can be published in its current form.

The authors have addressed all minor editorial requests.

Dr. Peter Kindgren
Umeå Plant Science Centre
Forest Genetics and Plant Physiology
Swedish University of Agricultural Sciences
Umeå,
Sweden

Dear Dr. Kindgren,

I am very pleased to accept your manuscript for publication in the next available issue of EMBO reports. Thank you for your contribution to our journal.

Your manuscript will be processed for publication by EMBO Press. It will be copy edited and you will receive page proofs prior to publication. Please note that you will be contacted by Springer Nature Author Services to complete licensing and payment information.

You may qualify for financial assistance for your publication charges - either via a Springer Nature fully open access agreement or an EMBO initiative. Check your eligibility: https://www.embopress.org/page/journal/14693178/authorguide#chargesguide

Should you be planning a Press Release on your article, please get in contact with embo_production@springernature.com as early as possible in order to coordinate publication and release dates.

If you have any questions, please do not hesitate to contact the Editorial Office. Thank you for your contribution to EMBO Reports.

Yours sincerely,

Yehu Moran
Editor
EMBO Reports

>>> Please note that it is EMBO Reports policy for the transcript of the editorial process (containing referee reports and your response letter) to be published as an online supplement to each paper. If you do NOT want this, you will need to inform the Editorial Office via email immediately. More information is available here: https://www.embopress.org/transparent-process#Review_Process

EMBO Press Author Checklist

Corresponding Author Name: Peter Kindgren	
Journal Submitted to: EMBO Reports	
Manuscript Number: EMBOR-2025-61946-T	

USEFUL LINKS FOR COMPLETING THIS FORM
The EMBO Journal - Author Guidelines
EMBO Reports - Author Guidelines
Molecular Systems Biology - Author Guidelines
EMBO Molecular Medicine - Author Guidelines

Reporting Checklist for Life Science Articles (updated January
This checklist is adapted from Materials Design Analysis Reporting (MDAR) Checklist for Authors. MDAR establishes a minimum set of requirements in transparent reporting in the life sciences (see Statement of Task: 10.31222/osf.io/9sm4x). Please follow the journal's guidelines in preparing your
Please note that a copy of this checklist will be published alongside your article.

Abridged guidelines for figures
1. Data
The data shown in figures should satisfy the following conditions:
- the data were obtained and processed according to the field's best practice and are presented to reflect the results of the experiments in an accurate and unbiased manner.
- ideally, figure panels should include only measurements that are directly comparable to each other and obtained with the same assay.
- plots include clearly labeled error bars for independent experiments and sample sizes. Unless justified, error bars should not be shown for technical
- if n<5, the individual data points from each experiment should be plotted. Any statistical test employed should be justified.
- Source Data should be included to report the data underlying figures according to the guidelines set out in the authorship guidelines on Data

2. Captions
Each figure caption should contain the following information, for each panel where they are relevant:
- a specification of the experimental system investigated (eg cell line, species name).
- the assay(s) and method(s) used to carry out the reported observations and measurements.
- an explicit mention of the biological and chemical entity(ies) that are being measured.
- an explicit mention of the biological and chemical entity(ies) that are altered/varied/perturbed in a controlled manner.
- the exact sample size (n) for each experimental group/condition, given as a number, not a range;
- a description of the sample collection allowing the reader to understand whether the samples represent technical or biological replicates (including how many animals, litters, cultures, etc.).
- a statement of how many times the experiment shown was independently replicated in the laboratory.
- definitions of statistical methods and measures:
  - common tests, such as t-test (please specify whether paired vs. unpaired), simple χ2 tests, Wilcoxon and Mann-Whitney tests, can be unambiguously identified by name only, but more complex techniques should be described in the methods section;
  - are tests one-sided or two-sided?
  - are there adjustments for multiple comparisons?
  - exact statistical test results, e.g., P values = x but not P values < x;
  - definition of 'center values' as median or average;
  - definition of error bars as s.d. or s.e.m.

Please complete ALL of the questions below.
Select "Not Applicable" only when the requested information is not relevant for your study.

Materials

Newly Created Materials	Information included in the manuscript?	In which section is the information available? (Reagents and Tools Table, Materials and Methods, Figures, Data Availability Section)
New materials and reagents need to be available; do any restrictions apply?	Yes	Used materials and reagents are listed in the Reagent and Tools Table. No restrictions apply

Antibodies	Information included in the manuscript?	In which section is the information available? (Reagents and Tools Table, Materials and Methods, Figures, Data Availability Section)
For **antibodies** provide the following information:<br>- Commercial antibodies: RRID (if possible) or supplier name, catalogue number and or/clone number<br>- Non-commercial: RRID or citation	Not Applicable	no antibodies were used

DNA and RNA sequences	Information included in the manuscript?	In which section is the information available? (Reagents and Tools Table, Materials and Methods, Figures, Data Availability Section)
**Short novel DNA or RNA including primers, probes**: provide the sequences.	Yes	Table EV1

Cell materials	Information included in the manuscript?	In which section is the information available? (Reagents and Tools Table, Materials and Methods, Figures, Data Availability Section)
**Cell lines:** Provide species information, strain. Provide accession number in repository **OR** supplier name, catalog number, clone number, and/**OR** RRID.	Yes	Reagents and Tools Table
**Primary cultures:** Provide species, strain, sex of origin, genetic modification status.	Not Applicable	
Report if the cell lines were recently **authenticated** (e.g., by STR profiling) and tested for mycoplasma contamination.	Not Applicable	

Experimental animals	Information included in the manuscript?	In which section is the information available? (Reagents and Tools Table, Materials and Methods, Figures, Data Availability Section)
**Laboratory animals or Model organisms:** Provide species, strain, sex, age, genetic modification status. Provide accession number in repository **OR** supplier name, catalog number, clone number, **OR** RRID.	Not Applicable	
**Animal observed in or captured from the field:** Provide species, sex, and age where possible.	Not Applicable	
Please detail **housing and husbandry conditions**.	Not Applicable	

Plants and microbes	Information included in the manuscript?	In which section is the information available? (Reagents and Tools Table, Materials and Methods, Figures, Data Availability Section)
**Plants:** provide species and strain, ecotype and cultivar where relevant, unique accession number if available, and source (including location for collected wild specimens).	Yes	Reagents and Tools Table and Materials and Methods section
**Microbes:** provide species and strain, unique accession number if available, and source.	Yes	Reagents and Tools Table and Materials and Methods section

Human research participants	Information included in the manuscript?	In which section is the information available? (Reagents and Tools Table, Materials and Methods, Figures, Data Availability Section)
If collected and within the bounds of privacy constraints report on age, sex and gender or ethnicity for all study participants.	Not Applicable	

Core facilities	Information included in the manuscript?	In which section is the information available? (Reagents and Tools Table, Materials and Methods, Figures, Data Availability Section)
If your work benefited from core facilities, was their service mentioned in the acknowledgments section?	Yes	Acknowledgments

Design

Study protocol	Information included in the manuscript?	In which section is the information available? (Reagents and Tools Table, Materials and Methods, Figures, Data Availability Section)
If study protocol has been **pre-registered, provide DOI in the manuscript**. For clinical trials, provide the trial registration number **OR** cite DOI.	Not Applicable	
Report the **clinical trial registration number** (at ClinicalTrials.gov or equivalent), where applicable.	Not Applicable	

Laboratory protocol	Information included in the manuscript?	In which section is the information available? (Reagents and Tools Table, Materials and Methods, Figures, Data Availability Section)
Provide DOI OR other citation details if **external detailed step-by-step protocols** are available.	Not Applicable	

Experimental study design and statistics	Information included in the manuscript?	In which section is the information available? (Reagents and Tools Table, Materials and Methods, Figures, Data Availability Section)
Include a statement about **sample size** estimate even if no statistical methods were used.	Yes	Figure legends
Were any steps taken to minimize the effects of subjective bias when allocating animals/samples to treatment (e.g. **randomization procedure**)? If yes, have they been described?	Not Applicable	
Include a statement about **blinding** even if no blinding was done.	Not Applicable	
Describe **inclusion/exclusion criteria** if samples or animals were excluded from the analysis. Were the criteria pre-established?<br><br>If sample or data points were omitted from analysis, report if this was due to attrition or intentional exclusion and provide justification.	Not Applicable	
For every figure, are **statistical tests** justified as appropriate? Do the data meet the assumptions of the tests (e.g., normal distribution)? Describe any methods used to assess it. Is there an estimate of variation within each group of data? Is the variance similar between the groups that are being statistically compared?	Yes	Figure legends and Material and methods

Sample definition and in-laboratory replication	Information included in the manuscript?	In which section is the information available? (Reagents and Tools Table, Materials and Methods, Figures, Data Availability Section)
In the figure legends: state number of times the experiment was **replicated** in laboratory.	Yes	Material and methods
In the figure legends: define whether data describe **technical or biological replicates**.	Yes	Figure legends

Ethics

Ethics	Information included in the manuscript?	In which section is the information available? (Reagents and Tools Table, Materials and Methods, Figures, Data Availability Section)
Studies involving **human participants**: State details of **authority granting ethics approval** (IRB or equivalent committee(s), provide reference number for approval.	Not Applicable	
Studies involving **human participants**: Include a statement confirming that **informed consent** was obtained from all subjects and that the experiments conformed to the principles set out in the WMA Declaration of Helsinki and the Department of Health and Human Services Belmont Report.	Not Applicable	
Studies involving **human participants:** For publication of **patient photos**, include a statement confirming that consent to publish was obtained.	Not Applicable	
Studies involving experimental **animals**: State details of **authority granting ethics approval** (IRB or equivalent committee(s), provide reference number for approval. Include a statement of compliance with ethical regulations.	Not Applicable	
Studies involving **specimen and field samples:** State if relevant **permits** obtained, provide details of authority approving study; if none were required, explain why.	Not Applicable	

Dual Use Research of Concern (DURC)	Information included in the manuscript?	In which section is the information available? (Reagents and Tools Table, Materials and Methods, Figures, Data Availability Section)
Could your study fall under dual use research restrictions? Please check biosecurity documents and list of **select agents and toxins** (CDC): https://www.selectagents.gov/sat/list.htm	Not Applicable	
If you used a select agent, is the security level of the lab appropriate and reported in the manuscript?	Not Applicable	
If a study is subject to dual use research of concern regulations, is the name of the **authority granting approval and reference number** for the regulatory approval provided in the manuscript?	Not Applicable	

Reporting

The MDAR framework recommends adoption of discipline-specific guidelines, established and endorsed through community initiatives. Journals have their own policy about requiring specific guidelines and recommendations to complement MDAR.

Adherence to community standards	Information included in the manuscript?	In which section is the information available? (Reagents and Tools Table, Materials and Methods, Figures, Data Availability Section)
State if relevant guidelines or checklists (e.g., **ICMJE, MIBBI, ARRIVE, PRISMA**) have been followed or provided.	Not Applicable	
For **tumor marker prognostic studies,** we recommend that you follow the **REMARK** reporting guidelines (see link list at top right). See author guidelines, under 'Reporting Guidelines'. Please confirm you have followed these guidelines.	Not Applicable	
For **phase II and III randomized controlled trials**, please refer to the **CONSORT** flow diagram (see link list at top right) and submit the CONSORT checklist (see link list at top right) with your submission. See author guidelines, under 'Reporting Guidelines'. Please confirm you have submitted this list.	Not Applicable	

Data Availability

Data availability	Information included in the manuscript?	In which section is the information available? (Reagents and Tools Table, Materials and Methods, Figures, Data Availability Section)
Have **primary datasets** been deposited according to the journal's guidelines (see 'Data Deposition' section) and the respective accession numbers provided in the Data Availability Section?	Not Applicable	
Were **human clinical and genomic datasets** deposited in a public access-controlled repository in accordance to ethical obligations to the patients and to the applicable consent agreement?	Not Applicable	
Are **computational models** that are central and integral to a study available without restrictions in a machine-readable form? Were the relevant accession numbers or links provided?	Not Applicable	
If publicly available data were reused, provide the respective **data citations in the reference list**.	Yes	Material and Methods section
